# Cohabitation and mental health: Is psychotropic medication use more common in cohabitation than marriage?

**DOI:** 10.1016/j.ssmph.2018.01.001

**Published:** 2018-02-02

**Authors:** Karen van Hedel, Pekka Martikainen, Heta Moustgaard, Mikko Myrskylä

**Affiliations:** a Max Planck Institute for Demographic Research, Rostock, Germany; b Population Research Unit, University of Helsinki, Helsinki, Finland; c Centre for Health Equity Studies (CHESS), Stockholm University and Karolinska Institutet, Stockholm, Sweden; d Department of Social Policy, London School of Economics and Political Science, London, UK

**Keywords:** Cohabitation, Finland, Living arrangements, Marital status, Mental health, Psychotropic medication use

## Abstract

Marriage is associated with better mental health. While research on the mental health of cohabiting individuals has increased in recent years, it has yielded mixed results thus far. We assessed whether the mental health of cohabiters is comparable to that of married individuals or those living alone using longitudinal data on psychotropic medication purchases. Panel data from an 11% random sample of the population residing in Finland for the years 1995 to 2007, with annual measurements of all covariates, were used. Ordinary least squares (OLS) models were applied to disentangle the relation between cohabitation and psychotropic medication purchases while controlling for relevant time-varying factors (age, education, economic activity, and number of children), and individual fixed effects (FE) models to further account for unobserved time-invariant individual factors. Our sample consisted of 63,077 men and 61,101 women aged 25 to 39 years in 1995. Descriptive results and the OLS model indicated that the likelihood of purchasing psychotropic medication was lowest for married individuals, higher for cohabiters, and highest for individuals living alone. This difference between cohabiting and married individuals disappeared after controlling for time-varying covariates (percent difference [% diff] for men: 0.3, 95% confidence interval [CI]: -0.0, 0.6; % diff for women: -0.2, 95% CI: -0.6, 0.2). Further controlling for unobserved confounders in the FE models did not change this non-significant difference between cohabiting and married individuals. The excess purchases of psychotropic medication among individuals living alone compared to those cohabiting decreased to 1.2 (95% CI: 1.0, 1.4) and 1.4 (95% CI: 1.1, 1.6) percentage-points in the fully-adjusted FE model for men and women, respectively. Similar results were found for all subcategories of psychotropic medication. In summary, these findings suggested that the mental health difference between cohabiting and married individuals, but not the difference between cohabiting individuals and those living alone, was largely due to selection.

## 1 Introduction

Married individuals generally enjoy better physical and mental health, lower mortality, and engage in healthier behaviors than unmarried individuals (Kiecolt-Glaser and Newton, 2001, Wood et al., 2007, Manzoli et al., 2007, Lamb et al., 2003, Kim and McKenry, 2002, Simon, 2002). For example, being continuously unmarried was linked to larger increases in depressive symptoms than being continuously married (Horwitz et al., 1996, Kim and McKenry, 2002, Marks and Lambert, 1998, Simon, 2002). Entering into a marriage decreased an individual’s depressive symptoms, whereas marital dissolution increased them (Lamb et al., 2003, Kim and McKenry, 2002, Simon, 2002, Wu and Hart, 2002). A study examining the association between divorce and psychotropic medication use among middle-aged Finns found that psychotropic medication use increased strongly before divorce, declined during the 1.5 years after divorce, and settled at a level 3 percentage point higher than that of continuously married individuals (Metsä-Simola & Martikainen, 2013). This finding was supported by Wade & Pevalin (2004) using longitudinal British data; separated or divorced individuals had a higher prevalence of poor mental health after union dissolution, but poor mental health was already reported before the dissolution. But having depressive symptoms did not affect the likelihood of an individual getting married (Lamb et al., 2003). Although the relationship between marriage and mental health has been studied extensively, less is known about the mental health effects of cohabitation.

### 1.1 (Non-marital) cohabitation

In the last few decades, non-marital cohabitation, hereafter referred to as cohabitation, has gained ground as a living arrangement in most high income countries (Heuveline and Timberlake, 2004, Kiernan, 2001, Seltzer, 2004). For example in Finland, 2.3 percent of the family population in 1970 involved a cohabiting couple (Official Statistics of Finland (OSF), 2015). Since then, the cohabitation rate has steadily increased and by 2015 almost 23 percent of Finnish families involved a cohabiting couple (Official Statistics of Finland (OSF), 2015). This rise in cohabitation has happened mostly at the expense of marriage. Whereas married couples were involved in 85.2 percent of Finnish families in 1970, this declined to 64.6 percent in 2015 (Official Statistics of Finland (OSF), 2015). A similar trend was found for other countries, although timing and the rate of increase may differ (Kalmijn, 2007, Kiernan, 1999, Sobotka and Toulemon, 2008). Nevertheless, Finland as well as the other Nordic countries remain to be notably different from other OECD countries with their high proportion of cohabiting couples (OECD, 2015).

Cohabitation may be chosen by individuals for several reasons, which may depend upon the temporal and geographical context; it could be considered an alternative for marriage, a prelude to marriage, or even an alternative to singlehood (Rindfuss & VandenHeuvel, 1990). These days, cohabitation is more often than before chosen as a long-standing alternative for marriage, as individuals consciously choose to spend their lives together but not to get married. Individuals are nowadays also more likely to choose to cohabitate before they marry, where the cohabitation itself acts as a trial marriage (Kulu & Boyle, 2010). This despite the fact that cohabiting relationships have become increasingly less likely to eventually progress into marriages (Kuo & Raley, 2016).

### 1.2 Similarities and differences between marriage and cohabitation as living arrangements

Being in a cohabiting relationship may offer the same benefits to an individual as those provided through marriage. For example, both marriage and cohabitation may provide social and economic advantages to an individual (Umberson & Williams, 1999). A partnership is an important source of social support, as it provides companionship and intimacy, plus an expanded social network, as an individual will also be connected to the social network of their partner (Amato, 2015). Furthermore, both married and cohabiting individuals could be better off economically; they may have two incomes at their disposal and thus be able to profit from economies of scale (Lerman, 2002). These social and economic advantages could in turn positively influence health. For example, a partner may encourage healthy behaviors (e.g., physical activity or healthy dietary habits) and discourage unhealthy ones (e.g., smoking or excessive alcohol consumption) (Umberson, 1987), and more economic resources could improve access to better quality health care. As most advantages of marriage are related to the presence of a partner, cohabitation may be able to offer similar benefits and as a result may have the same positive influence on health as marriage has.

However, cohabiting relationships may also differ from marriages in several ways (Nock, 1995). First, marriage comes with social norms and legal benefits and obligations, but cohabitation does not have these benefits. Even though the differences in legal benefits between cohabitation and marriage are likely to only have a minimum effect on the daily lives of Finns, marriage is still subjected to more legal regulations than cohabitation; e.g. there is a maintenance obligation for married partners, widowers pensions are only accessible for married partners, and cohabiting partners do not have an automatic inheritance right to their partner’s property (Mäenpää, 2015). Second, whereas economic resources are often managed jointly in a marriage, in a cohabiting relationship economic resources are often kept separate (Heimdal and Houseknecht, 2003, Lyngstad et al., 2011). However, the likelihood that a cohabiting couple pools their economic resources is higher if they intend to marry, than if they do not have any marriage intentions (Lyngstad et al., 2011). Lastly, cohabiting relationships are generally shorter in duration than marriages, and individuals in a cohabiting relationship seem to be less certain about their relationship than married individuals (Bumpass, Sweet, & Cherlin, 1991). This greater uncertainty in cohabiting relationships may result in lower levels of social support and social control, and having a marital partner may thus be more beneficial for an individual’s health than having a cohabiting partner. For example, cohabiting men have been found to be less likely to have had a health care visit than married men (Blumberg, Vahratian, & Blumberg, 2014). Also in line with this greater uncertainty surrounding their relationship, cohabiters are more likely to experience a union dissolution than married individuals (Moustgaard and Martikainen, 2009, Smock, 2000). Additionally, even if cohabiting individuals eventually marry, they are increasingly more likely to divorce than individuals who did not cohabit before they married (Axinn and Thornton, 1992, Lillard et al., 1995). But findings from a more recent study suggested that cohabitation helps avoid bad marriages, indicated by a lower likelihood to divorce among those who previously cohabited than those who directly married (Kulu & Boyle, 2010).

### 1.3 Cohabitation and mental health

Research on the mental health of cohabiting individuals as a distinct group, i.e. not grouped together with single individuals based on their legal marital status, has increased in recent years. Nonetheless, it has yielded mixed results thus far. Several studies have found that cohabiting individuals are worse off in terms of their mental health than married individuals. For example, Brown, Bulanda and Lee (2005) found that among the US population over age 50, cohabiting men reported significantly higher depression scores than married men, but cohabiting and married women reported similar depression scores. Using data from the National Survey of Families and Households in the United States, Brown (2000) found that cohabiters aged 19 and over reported significantly higher levels of depression than married individuals, even after controlling for several demographic factors. Using the same data, Marcussen (2005) found that even after controlling for socioeconomic resources, cohabiting individuals still reported higher levels of depression than married individuals. But when taking into account coping resources and relationship quality, that difference in depression between cohabiting and married individuals was reduced to non-significant. In addition, Willitts, Benzeval and Stansfeld (2004) found a gender difference in how cohabitation, marriage and mental health were related; cohabitation was more beneficial for the mental health of men, whereas marriage was more beneficial for the mental health of women.

In contrast, other studies have found no differences in the mental health of cohabiting and married individuals. For example, Ross (1995) showed in a study from the United States that the reported levels of depression were similar for married and cohabiting individuals aged 18 to 90 years. In a study of an American cohort of young adults (individuals who were 18, 21, or 24 years old at baseline) by Horwitz and White (1998), cohabitation was not associated with higher depression scores than marriage or singlehood, when controlling for several factors including previous depression. Using longitudinal data from American adolescents (students who were in Grades 7 through 12 at baseline), Amato (2015) found that cohabitation protected mental health in a similar way as marriage does whilst considering age, education, work hours, and parenthood. Using cross-sectional survey data for 30 to 64 year old Finns, Joutsenniemi, Martelin, Martikainen, Pirkola & Koskinen (2006) found no differences in depressive disorders, anxiety disorders and psychological distress for cohabiting and married individuals, when taking into account age, childhood circumstances, unemployment, and social support. Lindeman et al. (2000) found no significant difference in the likelihood of a major depressive episode between married and cohabiting men and women after adjusting for several socioeconomic and behavioral factors, using data for 15 to 75 year old Finns. Initially Wu, Penning, Pollard & Hart (2003) found a gradient in mental health using cross-sectional data for 20 to 64 year old Canadians; the mental health of cohabiters was worse than that of married individuals, but better than that of single individuals. However, this difference between cohabiting and married individuals became non-significant when taking into account other relevant factors, such as psychological and social resources, health risk factors, and demographic factors.

Most research examining the relationship between cohabitation and mental health has focused on depressive symptoms. However, other mental health or related outcomes have also been studied. For example, Horwitz and White (1998) found that cohabiting American young adults (individuals aged 18, 21, or 24 years at baseline) reported more alcohol problems than married young adults. Moreover, cohabiting men reported more alcohol problems than single men. Joutsenniemi et al. (2007) found that cohabitation, but also living alone, was associated with heavy drinking and alcohol dependence for men and women aged 30 to 54 years. Consistent with these two studies, Li, Wilsnack, Wilsnack & Kristjanson (2010) found an association of cohabitation with alcohol consumption in 19 countries, and with heavy drinking in 17 countries for men and women aged 18 to 65 years. Entering a marriage or cohabiting relationship reduced binge drinking and marijuana use among US young adults (men and women aged between 14 and 22 at first interview) (Duncan, Wilkerson, & England, 2006), where the reduction was larger for entering a marriage than a cohabiting relationship. Cohabitation has also been strongly associated with suicide and substance use disorders in many Nordic countries (Norlev et al., 2005, Qin et al., 2003). Furthermore, Musick and Bumpass (2012) found that for US adults under the age of 50 years at baseline in 1987–1988, marriage and cohabitation had similar effects on well-being. In contrast, Soons and Kalmijn (2009) found that well-being was higher among married young adults (individuals aged 18 to 44 years) than their cohabiting peers. But Soons, Liefbroer and Kalmijn (2009) found the well-being level of cohabiting young adults (aged 18 to 26 years at the start of their study) to be lower than that of married young adults, but higher than that of young adults not in a union.

Study results are likely to differ due to true differences in the policy and societal context of different populations. In different national settings and population subgroups, the trends and levels of cohabitation have evolved very differently. Also, the routes into cohabitation vary for older and younger participants. The meaning and consequences of cohabitation are thus likely to vary between study contexts. However, the lack of consistency in the evidence on the association between cohabitation and mental health may reflect differences in analyses and measurement, e.g. measurement of mental health, set of explanatory variables used, the type of data, and consequently the type of analysis used. Most studies have used cross-sectional study designs and did not take into account selection into different living arrangements. We will extend on that literature by taking into account selection in a longitudinal framework.

### 1.4 Aim of this study

The aim of this study was to assess how cohabiting young adults differ from married young adults and those living alone in terms of their psychotropic medication use (a proxy for mental health) in Finland – a Nordic country with comparatively early increase and high current levels of popularity of cohabitation. Annual longitudinal registration data linked to medication registries for men and women in Finland between 1995 and 2007 were used. As Finland is a vanguard country in the social acceptance of cohabitation, of which recent longitudinal data is available on cohabitation, results from the Finnish context may show the way for other Western countries in which cohabitation is still winning ground. Another unique contribution of this study is that we do not rely on self-reports of mental health and we have no loss to follow-up in our register-based panel. Furthermore, for more accurate causal inference we estimated an ordinary least squares model controlling for a set of observed time-varying confounding variables and an individual fixed effects model to additionally control for unobserved time-invariant confounders. For example, a fixed effects model has the advantage over a normal regression that it can control for all time-invariant factors, even if these are unmeasured.

## 2 Methods

### 2.1 Analytic sample

An 11% random sample representative of the population permanently residing in Finland at the end of any of the years 1995 to 2007 was used. Using a unique personal identification code, this sample was linked on an individual level to annual data from other official registries; namely the labor market data file and medication records. The latter contained all purchases of prescription medication with information on purchase dates as well as the amount and type of drug purchased. As we focused on Finnish young adults among whom cohabitation is common and even the norm before marriage, the sample was restricted to men and women aged 25 to 39 years in 1995. This sample of 63,077 men and 61,101 women was followed until the end of 2007 for sociodemographic factors and psychotropic medication purchases. During these 13 years, 2.4% and 1.6% of total observations were missing for men and women respectively, due to individuals not being part of the dwelling population of Finland in a specific year, i.e. they died or were (temporarily) abroad.

### 2.2 Purchased prescribed psychotropic medication

We used psychotropic medication purchases as a proxy measure for mental health. We focused on purchased prescribed psychotropic medication in general, but also by the following 4 subcategories: antidepressants, antipsychotics, antimanic agents, and anxiolytic/sedative/hypnotic (ASH) medication. The Anatomical Therapeutic Chemical codes (WHO Collaborating Centre for Drug Statistics Methodology, 2016) for these 4 subcategories are presented in Supplement Table A. Prescribed psychotropic medication was measured as having purchased at least one prescription of the above mentioned medications in a calendar year. The prevalence of the 4 subcategories possibly does not sum up to the prevalence of all psychotropic medication, as individuals may use multiple types of psychotropic medication at the same time.

### 2.3 Independent time-varying variables

Individuals were categorized into five groups based on their living arrangement status: (1) married individuals living with their partner; (2) cohabiting individuals living in the same dwelling with a partner of opposite sex, who was not a married spouse or a sibling and with whom the age difference did not exceed 15 years; (3) individuals living alone; (4) other living arrangements, such as individuals living with other adults, e.g. parents or housemates, or those living in institutions; and (5) individuals with an unknown living arrangement status. Age was included as 5-year age dummies to allow for the non-linear relationship between age and psychotropic medication. We also included year in the analyses to account for a possible time trend in the prescription of psychotropic medication.

We distinguished three categories of educational attainment based on the highest degree obtained by the individual: upper secondary or less education, lower tertiary education, and higher tertiary or more education. Regarding the number of children in the family, we differentiated between no children, 1 child, 2 children, and 3 or more children under the age of 18 years. Economic activity was divided into five categories; employed, unemployed, students and pupils, pensioners, and others (including the categories other, unknown, conscripts, and conscientious objectors).

We do not only expect that our explanatory factors, i.e. education, economic activity, and number of children, are related to psychotropic medication purchases, but also to cohabitation, or living arrangements in general. For example, the likelihood of cohabiting, as well as marriage, is higher among Finnish young adults with high education than those with lower levels of education (Jalovaara, 2012). Also, unemployed young adults are less likely to cohabit or marry than their employed peers, but young adults still in education were least likely to be in a union. Even though more than half of all children born in 2015 were born to married parents, 57 percent of first born children were born outside marriage (Official Statistics of Finland (OSF), 2016).

All variables were annually measured and treated as time-varying.

### 2.4 Statistical analysis

First, we analyzed the relationship between living arrangements and the purchase of prescribed psychotropic medication in an ordinary least squares model, only controlling for year and age (model 1). In model 2 we additionally controlled for education and economic activity, whereas in model 3 also the number of children was included as a time-varying variable. Number of children was added to the model separately from education and economic activity, as the association of mental health with parenthood is less clear than that of mental health with education and economic activity; there is conflicting empirical evidence on how parenthood may be associated with mental health. Next in models 4 to 6, we controlled for unobserved confounders by applying an individual fixed effects model on the relationship between living arrangements and prescribed psychotropic medication. Similar to models 1 to 3, model 4 included only year and age, model 5 additionally included education and economic activity, and model 6 also controlled for the number of children. Furthermore, we examined whether the relationship between living arrangements and psychotropic medication differed by the presence of children in the household. In these analyses, parenthood status was defined as having at least one child under the age of 18 years in the family, and was annually measured.

All analyses were done separately for men and women.

## 3 Results

In 1995, around a half of the men and women in our sample were married (42.7% of men, 52.9% of women, Table 1). About a fifth of all men and women cohabited with a partner (20.4% of men, 19.0% of women), and approximately another fifth was living alone (17.9% of men, 21.3% of women). The remaining men (19.1%) and women (6.8%) were in a different or unknown living arrangement. Overall, 5.4 percent of the men and 7.0 percent of the women had purchased psychotropic medication indicating mental health problems. For both men and women, a gradient in purchasing psychotropic medication by living arrangements was observed. The percentage of men and women with psychotropic medication purchases was lowest for those married (3.6% of men, 5.4% of women), slightly higher for those cohabiting (4.1% of men, 6.1% of women) and approximately double for those living alone (8.3% of men, 10.5% of women). This difference between married and cohabiting men and women was not statistically significant (Supplement Table B). In 2007, more men and women were married (51.6% of men, 55.9% of women) or living alone (22.9% of men, 26.0% of women), but less were cohabiting (16.0% of men, 15.1% of women) or had a different or unknown living arrangement (9.5% of men, 3.1% of women). The likelihood of purchasing psychotropic medication was higher in 2007 than in 1995 for both men and women (12.8% of men, 18.2% of women). This increase was statistically significant (Supplement Table B), and is probably largely due to the aging of our study sample. Again, the likelihood of purchasing psychotropic medication was lowest among married individuals (9.1% of men, 14.6% of women), slightly higher among cohabiting individuals (10.0% of men, 16.3% of women), and highest among individuals living alone (20.3% of men, 26.1% of women). For both 1995 and 2007, women were more likely than men to have purchased prescribed psychotropic medication in general, but also within each living arrangement status (Supplement Table B).

**Table 1:** Distribution of living arrangements and proportion of men and women with psychotropic medication purchases by living arrangement, 1995 and 2007.

	**1995**				**2007**			
	**Distribution**		**Purchased psychotropic medication**		**Distribution**		**Purchased psychotropic medication**	
	**No.**	**(%)**	**No.**	**(%)**	**No.**	**(%)**	**No.**	**(%)**
**Men, living arrangement**								
Married	25757	(42.7)	919	(3.6)	31105	(51.6)	2838	(9.1)
Cohabiting	12271	(20.4)	498	(4.1)	9641	(16.0)	962	(10.0)
Living alone	1079	(17.9)	893	(8.3)	13783	(22.9)	2804	(20.3)
Other	10657	(17.7)	815	(7.7)	4596	(7.6)	859	(18.7)
Unknown	833	(1.4)	112	(13.5)	1152	(1.9)	263	(22.8)
Total	60277		3237	(5.4)	60277		7726	(12.8)
**Women, living arrangement**								
Married	31429	(52.9)	1685	(5.4)	33185	(55.9)	4848	(14.6)
Cohabiting	11256	(19.0)	685	(6.1)	8935	(15.1)	1453	(16.3)
Living alone	12642	(21.3)	1325	(10.5)	15412	(26.0)	4026	(26.1)
Other	3669	(6.2)	382	(10.4)	1347	(2.3)	372	(27.6)
Unknown	375	(0.6)	47	(12.5)	492	(0.8)	129	(26.2)
Total	59371		4124	(7.0)	59371		10828	(18.2)

Notes*:* For descriptive purposes, this table included men and women aged 25 to 39 years in 1995 and who had data available for both 1995 and 2007. The proportion of men and women with specific subcategories of psychotropic medication purchases can be found in Supplement Table C.

### 3.1 The ordinary least squares (OLS) models

**Fig. 1:**
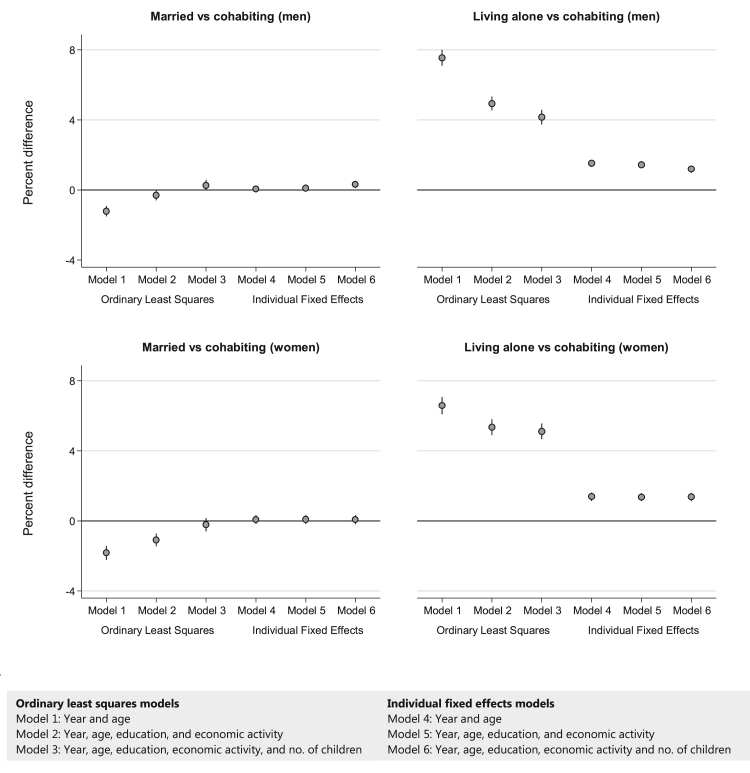
Percent differences in the likelihood of psychotropic medication purchases for different living arrangements of men and women aged 25-39 years in 1995 followed up to 2007. Notes: Coefficients from the OLS and FE models were multiplied by 100 to present percent changes in the likelihood of purchasing psychotropic medication. The error bars represent 95% confidence intervals. All analyses were controlled for 5-year age groups and year. Models were additionally controlled for educational attainment, economic activity, and number of children in the family, where mentioned. Full information on point estimates and 95% confidence intervals can be found in Supplement Table D and corresponding predicted probabilities in Supplement Table E.

Results from the first OLS model, only controlling for year and age, indicated that married men (Fig. 1, top left) were 1.2 percentage points less likely to have purchased psychotropic medication than cohabiting men (percent difference compared to cohabiting men [% diff]: -1.2, 95% confidence interval [CI]: -1.5, -0.9, Supplement Table D). Of the married men, 5.2 percent purchased psychotropic medication, compared to 6.4 percent of cohabiting men and 14.0 percent of men living alone (Supplement Table E). Men living alone (top right) were thus 7.6 percentage points (95% CI: 7.1, 8.0) more likely to purchase psychotropic medication than cohabiting men. After controlling for education and economic activity (model 2), the differences in likelihood of prescribed psychotropic medication became smaller: the advantage of married men attenuated to 0.3 (% diff: -0.3, 95% CI: -0.6, -0.0), whereas the disadvantage of men living alone attenuated to 4.9 (95% CI: 4.5, 5.3). Further controlling for number of children resulted in a difference of 0.3 percentage points between married and cohabiting men (% diff: 0.3, 95% CI: -0.0, 0.6), whereas it further reduced the difference between cohabiting men and those living alone to 4.2 (95% CI: 3.7, 4.6).

For women, we found a very similar pattern. Married women had an advantage of 1.8 percentage points (% diff: -1.8, 95% CI: -2.2, -1.4) in terms of their psychotropic medication purchases compared to cohabiting women (Fig. 1, bottom left). This advantage attenuated to 1.1 (% diff: -1.1, 95% CI: -1.5, -0.7) when controlling for education and economic activity (model 2), and was even further attenuated to non-significant (% diff: -0.2, 95% CI: -0.6, 0.2) when we also controlled for number of children (model 3). Comparing cohabiting women with women living alone (bottom right), we found that women living alone were initially 6.6 percentage points (95% CI: 6.1, 7.1) more likely to have purchased psychotropic medication (model 1). This difference was reduced to 5.4 (95% CI: 4.9, 5.8) when controlling for education and economic activity (model 2), and further reduced to 5.1 (95% CI: 4.7, 5.6) when additionally controlling for number of children (model 3).

### 3.2 Individual fixed effects models

In model 4, a fixed effects model only controlling for age and year (Fig. 1), no significant difference was found in purchased psychotropic medication prescriptions for married and cohabiting men (% diff: 0.1, 95% CI: -0.1, 0.3). Controlling for education and economic activity (model 5) hardly affected this already non-significant difference between married and cohabiting men (% diff: 0.1, 95% CI: -0.1, 0.3). When we additionally controlled for the number of children (model 6), married men were 0.3 percentage points (95% CI: 0.1, 0.5) more likely to purchase psychotropic medication than cohabiting men. The fixed effects models for living alone versus cohabiting men showed that men living alone were more likely to have purchased psychotropic medication (model 4, % diff: 1.5, 95% CI: 1.3, 1.7), although controlling for education and economic activity (model 5, % diff: 1.5, 95% CI: 1.3, 1.6) and subsequently number of children (model 6, % diff: 1.2, 95% CI: 1.0, 1.4) attenuated some of this disadvantage.

For women, we again found a similar pattern. The fixed effects models (models 4 to 6) for married versus cohabiting women showed that there was no statistically significant difference between purchased psychotropic medication of married and cohabiting women. Contrary to the results for men, controlling for number of children (model 6) did not affect the significance of the estimate of married versus cohabiting women. The fixed effects models (models 4–6) for living alone versus cohabiting women showed that women living alone were more likely to have purchased psychotropic medication than cohabiting women. This finding held after controlling for education and economic activity, and subsequently number of children.

### 3.3 Results by parenthood status

Whereas controlling for number of children hardly affected the overall estimates for women, it did affect those for men. Hence we stratified the analysis by parenthood, defined as having at least one child under the age of 18 years in the family and measured annually, to see whether the association between living arrangements and purchased psychotropic medication differed for individuals with and without children in the family.

**Fig. 2:**
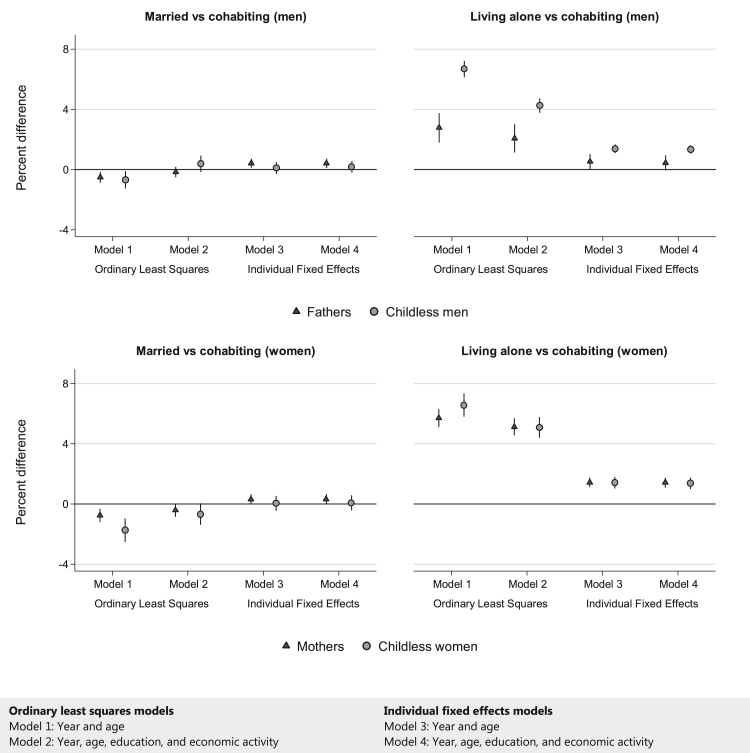
Percent differences in the likelihood of psychotropic medication purchases for different living arrangements comparing parents with childless men and women aged 25–39 years in 1995 followed up to 2007. Notes: Coefficients from the OLS and FE models were multiplied by 100 to present percent changes in the likelihood of purchasing psychotropic medication. The error bars represent 95% confidence intervals. All analyses were controlled for 5-year age groups and year. Models were additionally controlled for educational attainment and economic activity, where mentioned. Men and women were defined as fathers and mothers respectively, when they had at least one child under the age of 18 years living in their family. Full information on point estimates and 95% confidence intervals can be found in Supplement Table F and corresponding predicted probabilities in Supplement Table G.

The difference in psychotropic medication purchases between married and cohabiting men was similar for fathers and men without children in all models (Fig. 2, top left). However, the difference in psychotropic medication purchases between cohabiting men and men living alone was in general larger for men without children than for fathers. This difference between childless men and fathers was statistically significant in all models, including the fixed effects model controlling for education and economic activity (model 4, Supplement Table F). The estimated difference in psychotropic medication purchases between married and cohabiting women (Fig. 2, bottom left) seemed higher for mothers than for women without children, however these differences were not statistically significant (Supplement Table F). Although the difference in psychotropic medication purchases between cohabiting women and women living alone was larger for women without children than mothers in model 1, this difference disappeared in the other models (Supplement Table F).

### 3.4 Living arrangements and subcategories of psychotropic medication

**Fig. 3:**
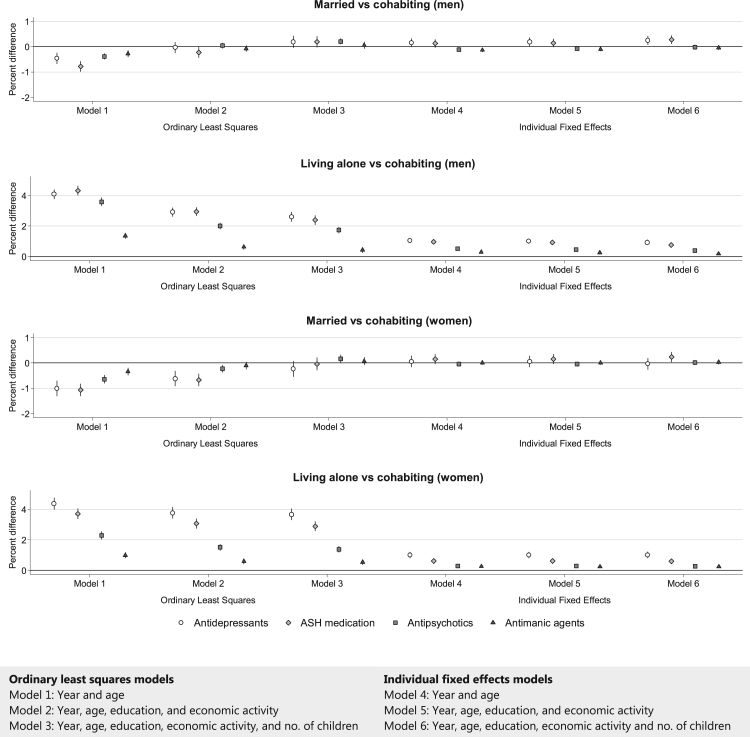
Percent differences in the likelihood of psychotropic medication purchases by subcategory for different living arrangements of men and women aged 25–39 years in 1995 followed up to 2007. Notes: Coefficients from the OLS and FE models were multiplied by 100 to present percent changes in the likelihood of purchasing psychotropic medication. The error bars represent 95% confidence intervals. All analyses were controlled for 5-year age groups and year. Models were additionally controlled for educational attainment, economic activity and number of children in the family, where mentioned. Full information on point estimates and 95% confidence intervals can be found in Supplement Table H, and corresponding predicted probabilities in Supplement Table I.

For both men and women, we found a similar pattern for all 4 subcategories of psychotropic medication, which corresponded with that for psychotropic medication in general. In the OLS model controlling for year and age (Fig. 3, model 1), married men and women had a lower likelihood of purchasing all subcategories of psychotropic medication than cohabiting men and women. After controlling for education, economic activity, and number of children (model 3), we only found a disadvantage in terms of antipsychotics for married men (% diff: 0.2, 95% CI: 0.1, 0.3, Supplement Table H). In the fixed effects models controlling for all time-varying covariates, married men were more likely to purchase antidepressants (% diff: 0.2, 95% CI: 0.1, 0.4) and ASH medication (% diff: 0.3, 95% CI: 0.1, 0.4), but we did not find this disadvantage for antipsychotics (% diff: -0.0, 95% CI: -0.1, 0.1) or antimanic agents (% diff: -0.0, 95% CI: -0.1, 0.0). We also did not find any difference between married and cohabiting women in the fixed effects models. Men and women living alone were worse off than cohabiting men and women in all models; i.e. they were more likely to have purchased any of the subcategories of psychotropic medication.

## 4 Discussion

The descriptive results and the ordinary least squares model indicated that the likelihood of purchasing prescribed psychotropic medication (a proxy for mental health problems) was lowest for married individuals, higher for cohabiting individuals, and highest for individuals living alone. After controlling for time-varying factors in the ordinary least squares model, the difference in likelihood of purchasing psychotropic medication between cohabiting and married men and women disappeared. Further controlling for unobserved confounders by applying an individual fixed effects model did not change the non-significant difference in the likelihood of purchasing psychotropic medication between cohabiting and married individuals. However, it did decrease the difference between cohabiting individuals and those living alone. A similar pattern was found for men and women with and without children in the family, as well as for the 4 subcategories of psychotropic medication.

### 4.1 Methodological considerations

A major strength of this study was the use of data on purchases of prescribed psychotropic medications from official medication registries for young adults in Finland, a Nordic country with high current levels of cohabitation. This allowed us to improve understanding of how living arrangements and mental health were related among young adults in present times, where cohabitation has become prevalent, by relying on longitudinal data with low levels of loss to follow-up, i.e. 2% of yearly observations were missing due to mortality or emigration. This better understanding of the mental health implications of cohabitation is especially important as cohabitation rates will most likely continue to rise in the future.

In this study, fixed effects models were used. The fixed effects results reflect only within-person variation, as individuals are treated as their own controls, and it thus does not rely on information on between-person comparisons. Selection may play an important role in the relationship between cohabitation and mental health, but these fixed effects models controlled for all unobserved time-invariant confounding. However, we recognize the importance of a better understanding of the size of this selection and therefore recommend studies studying the life-course of individuals to estimate the contribution of (mental) health early in life, as well as other demographic and socioeconomic factors, in explaining the likelihood of them cohabiting.

In this study, we used ordinary least squares (OLS) models to analyze the relationship between living arrangements and psychotropic medication purchases for several reasons. A major advantage of using an OLS model is the ease of interpreting its coefficients. Also, when estimating fixed effects (FE) models, a substantial proportion of the study sample has to experience change in the exposure. However, study subjects who do not experience change in the outcome are excluded from logistic FE models, whereas they would still be included in linear FE models (Allison, 2009). Therefore a substantially larger portion of the sample was retained when using linear FE models. Nonetheless, the results of ordinary logistic models and FE logistic models are presented in the supplemental materials (Supplement Figures A-C and Supplement Tables J-L). The logistic models produced qualitatively similar results to those of the OLS models, and they substantiate our main conclusion that psychotropic medication purchases are not more common in cohabitation than in marriage. However, we find larger odds of purchasing psychotropic medication for married individuals, when compared with cohabiting individuals in the FE models. This is likely due to the selectivity of the sample used in these models. Our model uses within-individual variation in living arrangements and psychotropic medication purchases to estimate the difference in medication purchases between various living arrangement states, averaging over all observation years. Thus our model does not pick up potential short-term fluctuations in psychotropic medication purchases around the change of the living arrangement status. Further research could study in more detail the short-term mental health effects of specific changes in living arrangements.

We used purchases of prescribed psychotropic medication, as this objective measure could be linked on an individual basis for all permanent residents of Finland. While purchased psychotropic medication is a good indicator of poorer mental health, it is not a perfect one; not all individuals with need for psychotropic medication are prescribed these medications, whereas there are also individuals prescribed these medication, without being diagnosed with a psychiatric disorder (Mark, 2010, Sihvo et al., 2008). It is possible that this unmet need for psychotropic medication differs by living arrangement (Hämäläinen et al., 2009); married and cohabiting individuals may be more likely to benefit from support from their partner in helping them with seeking help for their mental health problems than individuals living alone. If individuals living alone would have a greater unmet need for psychotropic medication, we are likely to have underestimated their psychotropic medication purchases, and thus also their disadvantage compared with married and cohabiting individuals. Hence, using purchased prescribed psychotropic medication to draw conclusions on overall mental health needs to be done carefully. To the extent that these measurement biases are time-invariant, we are likely to overcome these as the fixed effects approach assesses within-individual variability and thus is not affected by individual time-invariant factors for seeking or adhering to treatment.

### 4.2 Interpretation

Consistent with findings from previous studies concerning young adults (Soons and Kalmijn, 2009, Soons et al., 2009), we found that married men and women had better mental health than men and women with other living arrangements in the ordinary least squares models. However, selection into marriage and cohabitation may play an important role in this finding. After controlling for observed time-varying and unobserved time-invariant factors, we found no difference in the likelihood of purchasing psychotropic medication between married and cohabiting men and women. This finding is in line with other studies (Amato, 2015, Horwitz and White, 1998, Musick and Bumpass, 2012), in which no differences in mental health between married and cohabiting young adults were found. In addition, controlling for observed time-varying and unobserved time-invariant factors strongly attenuated the difference in purchasing psychotropic medication between cohabiting individuals and those living alone. This finding indicated that even after controlling for some selection by accounting for unobserved time-invariant factors, the disadvantage of individuals living alone as compared to cohabiting individuals remained.

The lack of consistency in evidence on the association between cohabitation and mental health may reflect differences in study contexts and designs. Differences in results may be a result of differences in the policy and societal context of the studies; cohabitation may be more likely to have mental health effects similar to those of marriage in countries where cohabitation is more common and possibly better regulated (Soons & Kalmijn, 2009). Furthermore, many studies used self-reported mental health, but those results may be biased due to the subjective nature of this measure. In addition, different measurements of mental health (e.g., clinical depression, antidepressants use) may lead to different results. For example, compared with marriage, cohabitation was associated with higher alcohol consumption and lower well-being in all previously mentioned studies (Duncan et al., 2006, Joutsenniemi et al., 2007, Li et al., 2010, Norlev et al., 2005, Qin et al., 2003, Soons and Kalmijn, 2009, Soons et al., 2009), but one (Musick & Bumpass, 2012). However, results were less clear regarding depressive symptoms. To ensure that an association between cohabitation and mental health is due to cohabitation in itself, various confounding variables should be included. But studies may differ significantly in how well such potential confounders are measured and accounted for. As we find a difference in the crude models but not the adjusted models, the difference in mental health between cohabiting and married individuals found in other studies could be a result of inadequate adjustment. Another possible explanation is the type of data, and consequently the type of analysis used. For example, some cross-sectional studies found higher reported depression levels for cohabiting individuals (Brown, 2000, Brown et al., 2005), but other studies, both cross-sectional and longitudinal, found no differences between married and cohabiting individuals with respect to depressive symptoms (Amato, 2015, Horwitz and White, 1998, Joutsenniemi et al., 2006, Lindeman et al., 2000, Marcussen, 2005, Ross, 1995, Wu et al., 2003). The relationship between marriage (or cohabitation) and mental health may be subject to selection (Wu et al., 2003), i.e. whether an individual marries (or cohabits) may depend upon their prior mental health, which cannot be properly dealt with in cross-sectional research. A longitudinal approach is thus required; one that controls for selection and possibly other unmeasured confounders.

As discussed earlier, we found that married and cohabiting men do not differ in their likelihood of purchasing psychotropic medication when we controlled for unobserved time-invariant factors, as well as the observed time-varying factors education and economic activity. However, after additionally controlling for number of children in the model, married men were more likely to purchase psychotropic medication than cohabiting men. Although the exact relationship between having children and mental health, or well-being in general, remains unclear, there is some temporary increase in happiness of parents after the birth of their child(ren) (Myrskylä & Margolis, 2014). Also in our data, having children was associated with lower levels of psychotropic medication purchases in both the ordinary least squares and the fixed effects specifications. As a result, accounting for fatherhood in our regression models explained − because married men are more likely to have children than cohabiting men−the remaining lower medication use among the married as opposed to the cohabiting. The effect of children on mental health was specific, i.e. it was only found for antidepressants and ASH medication, and not for antipsychotics and antimanic agents. Whereas antidepressants and ASH medication are often used for less severe mental health problems such as psychological distress or sleeping problems, antimanic and antipsychotic drugs are prescribed for more severe, chronic disorders. The effects of the latter are more likely to affect the likelihood of being in a partnership in general, rather than the choice between marriage and cohabitation. In contrast, among women controlling for the number of children in the family only slightly affected the estimates for purchasing psychotropic medication; the change was small and the difference between married and cohabiting women remained non-significant. In addition, the estimates of cohabiting men versus men living alone seemed smaller for fathers than for men without children, although they were not significantly different. Yet, this suggests that parenthood status may be particularly associated with better mental health for men, as having children is more common among men in a cohabiting union than those living alone.

## 5 Conclusion

Overall, our results showed that both men and women who are cohabiting had worse mental health than married men and women. However, controlling for observed and unobserved differences between cohabiting and married individuals indicated that the crude difference was likely due to differentials in selection processes into marriage and cohabitation. However, men and women living alone remained to be disadvantaged, suggesting that selection into partnership does not fully explain the mental health disadvantage of individuals living alone. Therefore, adequate interventions and policies to improve the mental health of individuals living alone may be needed. Our results nevertheless suggest that cohabitation provides similar mental health benefits as marriage in a context where cohabitation is the norm, at least for young adults.
