# Supplementary material for: Cohabitation and mental health: Is psychotropic medication use more common in cohabitation than marriage?
Source: SSM Popul Health. 2018 Feb 2;4:244–53. doi: 10.1016/j.ssmph.2018.01.001 (PMC5976833; doi:10.1016/j.ssmph.2018.01.001)
Supplement: Supplementary file 1 — Supplementary material [file mmc1.docx]

**Cohabitation and mental health: Is psychotropic medication use more common in cohabitation than marriage?**

**Supplemental materials**

**Table A**―Psychotropic medication and their Anatomical Therapeutic Chemical (ATC) codes

| **Subcategories** | **Anatomical Therapeutic Chemical (ATC) codes** |
| --- | --- |
| Antidepressants | N06A |
| Antipsychotics | N05A |
| Antimanic agents | N05AX12, N05AH5, N03AF01, N05AN01, N05AH03, N03AG01, N05AE04 |
| Anxiolytic/sedative/hypnotic (ASH) medication | N05B, N05C |
| **All psychotropic medication** | All of the above |

**Table B**―Estimated proportion of prescription of psychotropic medication by living arrangement for men and women aged 25 to 39 in 1995 for 1995 and 2007

|  | **1995** | | | **2007** | | | **Over time** |
| --- | --- | --- | --- | --- | --- | --- | --- |
|  | **Estimated proportion of psychotropic medication (95% CI)** | **Comparison of the estimated proportions of living arrangements with cohabiting – p value** | **Comparison of the estimated proportions between men and women by living arrangement – p value** | **Estimated proportion of psychotropic medication (95% CI)** | **Comparison of the estimated proportions of living arrangements with cohabiting – p value** | **Comparison of the estimated proportions between men and women by living arrangement – p value** | **Comparison of the estimated proportions between 1995 and 2007 –  p value** |
| **Men, living arrangement** |  |  |  |  |  |  |  |
| Married | 3.7 (3.5, 4.0) | 0.0029 | <0.0001 | 9.1 (8.8, 9.4) | 0.0136 | <0.0001 | <0.0001 |
| Cohabiting | 4.4 (4.0, 4.8) | Ref. | <0.0001 | 10.0 (9.4, 10.6) | Ref. | <0.0001 | <0.0001 |
| Living alone | 9.5 (9.0, 10.1) | <0.0001 | 0.0002 | 20.3 (19.7, 21.0) | <0.0001 | <0.0001 | <0.0001 |
| Other | 8.5 (8.0, 9.0) | <0.0001 | 0.0002 | 18.7 (17.6, 19.8) | <0.0001 | <0.0001 | <0.0001 |
| Unknown | 13.8 (11.8, 16.1) | <0.0001 | 0.2783 | 22.8 (20.5, 25.3) | <0.0001 | 0.1473 | <0.0001 |
| Total | 5.9 (5.8, 6.1) |  | <0.0001 | 12.8 (12.6, 13.1) |  | <0.0001 | <0.0001 |
| **Women, living arrangement** |  |  |  |  |  |  |  |
| Married | 5.4 (5.2, 5.7) | 0.0013 |  | 14.6 (14.2, 15.0) | 0.0001 |  | <0.0001 |
| Cohabiting | 6.3 (5.8, 6.7) | Ref. |  | 16.3 (15.5, 17.0) | Ref. |  | <0.0001 |
| Living alone | 11.0 (10.4, 11.5) | <0.0001 |  | 26.1 (25.4, 26.8) | <0.0001 |  | <0.0001 |
| Other | 10.6 (9.7, 11.6) | <0.0001 |  | 27.6 (25.3, 30.1) | <0.0001 |  | <0.0001 |
| Unknown | 11.8 (9.1, 15.1) | <0.0001 |  | 26.2 (22.5, 30.3) | <0.0001 |  | <0.0001 |
| Total | 7.2 (7.0, 7.4) |  |  | 18.2 (17.9, 18.6) |  |  | <0.0001 |

Notes: Ref.: reference category. 95% confidence intervals in brackets.

**Table C**―Distribution of living arrangements and proportion of men and women with purchases of psychotropic medication by living arrangement, 1995 and 2007

|  | **Distribution** | | **Purchased  psychotropic medication** | **Subcategories of purchased psychotropic medication** | | | |
| --- | --- | --- | --- | --- | --- | --- | --- |
|  |  |  |  | **Anti-depressants** | **ASH medication** | **Antipsychotics** | **Antimanic agents** |
|  | **No.** | **(%)** | **(%)** | **(%)** | **(%)** | **(%)** | **(%)** |
| **Men** |  |  |  |  |  |  |  |
| **1995, living arrangement** |  |  |  |  |  |  |  |
| Married | 25 757 | 42.7 | 3.6 | 1.5 | 2.5 | 0.5 | 0.4 |
| Cohabiting | 12 271 | 20.4 | 4.1 | 1.7 | 2.6 | 0.7 | 0.5 |
| Living alone | 1079 | 17.9 | 8.3 | 3.6 | 5.2 | 2.6 | 1.1 |
| Other | 10 657 | 17.7 | 7.7 | 2.6 | 4.2 | 3.4 | 1.1 |
| Unknown | 833 | 1.4 | 13.5 | 4.7 | 7.9 | 6.5 | 2.0 |
| Total | 60 277 | 100 | 5.4 | 2.2 | 3.4 | 1.5 | 0.7 |
| **2007, living arrangement** |  |  |  |  |  |  |  |
| Married | 31 105 | 51.6 | 9.1 | 5.6 | 5.3 | 0.9 | 0.9 |
| Cohabiting | 9641 | 16.0 | 10.0 | 5.9 | 5.9 | 1.2 | 1.1 |
| Living alone | 13 783 | 22.9 | 20.3 | 11.6 | 12.1 | 5.9 | 3.2 |
| Other | 4596 | 7.6 | 18.7 | 9.1 | 9.6 | 7.9 | 4.9 |
| Unknown | 1152 | 1.9 | 22.8 | 10.7 | 13.5 | 13.6 | 7.3 |
| Total | 60 277 | 100 | 12.8 | 7.4 | 7.4 | 2.9 | 1.9 |
| **Women** |  |  |  |  |  |  |  |
| **1995, living arrangement** |  |  |  |  |  |  |  |
| Married | 31 429 | 52.9 | 5.4 | 2.6 | 3.3 | 0.8 | 0.5 |
| Cohabiting | 11 256 | 19.0 | 6.1 | 2.7 | 3.9 | 1.1 | 0.7 |
| Living alone | 12 642 | 21.3 | 10.5 | 5.2 | 6.7 | 2.6 | 0.9 |
| Other | 3669 | 6.2 | 10.4 | 3.7 | 5.3 | 4.7 | 1.9 |
| Unknown | 375 | 0.6 | 12.5 | 4.0 | 6.4 | 6.4 | 2.9 |
| Total | 59 371 | 100 | 7.0 | 3.2 | 4.3 | 1.5 | 0.7 |
| **2007, living arrangement** |  |  |  |  |  |  |  |
| Married | 33 185 | 55.9 | 14.6 | 9.9 | 7.6 | 1.3 | 1.1 |
| Cohabiting | 8935 | 15.1 | 16.3 | 10.8 | 8.8 | 2.1 | 1.5 |
| Living alone | 15 412 | 26.0 | 26.1 | 17.6 | 14.6 | 5.4 | 3.1 |
| Other | 1347 | 2.3 | 27.6 | 13.1 | 14.6 | 13.0 | 8.6 |
| Unknown | 492 | 0.8 | 26.2 | 15.0 | 15.7 | 14.2 | 10.0 |
| Total | 59 371 | 100 | 18.2 | 12.1 | 9.8 | 2.8 | 1.9 |

Notes*:* For descriptive purposes, this table included men and women aged 25 to 39 years in 1995 and who had data available for both 1995 and 2007. ASH medication: anxiolytic/sedative/hypnotic medication. Anatomical Therapeutic Chemical (ATC) codes available in Table A.

**Table D**―Percent differences in psychotropic medication for different living arrangements of men and women aged 25-39 years in 1995, for 1995 to 2007

|  | **Ordinary Least Squares** | | | **Individual Fixed Effects** | | |
| --- | --- | --- | --- | --- | --- | --- |
|  | **Model 1** | **Model 2** | **Model 3** | **Model 4** | **Model 5** | **Model 6** |
| The models controlled for: | Year, age | Year, age, education, economic activity | Year, age, education, economic activity, no. of children | Year, age | Year, age, education, economic activity | Year, age, education, economic activity, no. of children |
| **Men, Sample size (No. of obs.)** | 63077 (800650) | 63077 (800650) | 63077 (800650) | 63077 (800650) | 63077 (800650) | 63077 (800650) |
| **Living arrangement** |  |  |  |  |  |  |
| Married | -1.2 (-1.5, -0.9) | -0.3 (-0.6, -0.0) | 0.3 (-0.0, 0.6) | 0.1 (-0.1, 0.3) | 0.1 (-0.1, 0.3) | 0.3 (0.1, 0.5) |
| Cohabiting | Ref. | Ref. | Ref. | Ref. | Ref. | Ref. |
| Living alone | 7.6 (7.1, 8.0) | 4.9 (4.5, 5.3) | 4.2 (3.7, 4.6) | 1.5 (1.3, 1.7) | 1.5 (1.3, 1.6) | 1.2 (1.0, 1.4) |
| Other | 7.0 (6.4, 7.6) | 2.0 (1.5, 2.5) | 1.1 (0.6, 1.7) | 0.8 (0.6, 1.0) | 0.7 (0.5, 1.0) | 0.5 (0.2, 0.7) |
| Unknown | 11.2 (9.8, 12.5) | -0.6 (-1.8, 0.7) | -1.4 (-2.7, 0.1) | -0.7 (-1.1, -0.3) | -1.1 (-1.5, -0.6) | -1.4 (-1.8, -0.9) |
| **Educational attainment** |  |  |  |  |  |  |
| Higher tertiary or more |  | Ref. | Ref. |  | Ref. | Ref. |
| Lower tertiary |  | -1.2 (-1.7, -0.7) | -1.2 (-1.7, 0.7) |  | -1.6 (-2.3, -0.9) | -1.6 (-2.3, -0.9) |
| Upper secondary |  | -1.2 (-1.7, -0.7) | -1.2 (-1.7, -0.7) |  | -1.3 (-1.9, -0.6) | -1.3 (-2.0, -0.7) |
| Compulsory |  | -0.5 (-1.0, 0.1) | -0.5 (-1.0, 0.1) |  | -0.9 (-1.8, 0.0) | -0.9 (-1.8, 0.0) |
| **Economic activity** |  |  |  |  |  |  |
| Employed |  | Ref. | Ref. |  | Ref. | Ref. |
| Unemployed |  | 7.0 (6.5, 7.4) | 6.9 (6.5, 7.3) |  | 0.6 (0.4, 0.8) | 0.6 (0.4, 0.8) |
| Students and pupils |  | 7.5 (6.8, 8.3) | 7.5 (6.7, 8.3) |  | 1.3 (0.9, 1.6) | 1.3 (0.9, 1.6) |
| Pensioners |  | 45.2 (43.6, 46.7) | 45.1 (43.5, 46.6) |  | 10.9 (10.4, 11.4) | 10.9 (10.4, 11.3) |
| Others |  | 7.6 (6.9, 8.3) | 7.5 (6.8, 8.2) |  | 2.3 (2.0, 2.6) | 2.3 (1.9, 2.6) |
| **No. of children < 18 years** |  |  |  |  |  |  |
| No children |  |  | 1.4 (1.1, 1.8) |  |  | 0.6 (0.4, 0.8) |
| 1 child |  |  | Ref. |  |  | Ref. |
| 2 children |  |  | -0.4 (-0.7, -0.1) |  |  | -0.2 (-0.4, -0.0) |
| 3 or more children |  |  | -0.6 (-0.9, -0.2) |  |  | -0.2 (-0.4, 0.0) |
| **Constant** | 1.7 (1.4, 2.0) | 1.2 (0.7, 1.8) | 0.5 (-0.1, 1.1) | 5.0 (4.7, 5.3) | 5.6 (4.9, 6.2) | 5.3 (4.6, 5.9) |
| **Women, Sample size (No. of obs.)** | 61101 (781368) | 61101 (781368) | 61101 (781368) | 61101 (781368) | 61101 (781368) | 61101 (781368) |
| **Living arrangement** |  |  |  |  |  |  |
| Married | -1.8 (-2.2, -1.4) | -1.1 (-1.5, -0.7) | -0.2 (-0.6, 0.2) | 0.1 (-0.2, 0.3) | 0.1 (-0.2, 0.3) | 0.1 (-0.2, 0.3) |
| Cohabiting | Ref. | Ref. | Ref. | Ref. | Ref. | Ref. |
| Living alone | 6.6 (6.1, 7.1) | 5.4 (4.9, 5.8) | 5.1 (4.7, 5.6) | 1.4 (1.2, 1.6) | 1.4 (1.1, 1.6) | 1.4 (1.1, 1.6) |
| Other | 8.2 (7.1, 9.4) | 1.0 (0.0, 1.9) | -0.3 (-1.3, 0.7) | 1.1 (0.6, 1.5) | 1.1 (0.6, 1.5) | 1.0 (0.6, 1.5) |
| Unknown | 6.7 (4.6, 8.9) | -6.8 (-8.7, -4.9) | -8.3 (-10.2, -6.3) | -2.3 (-3.2, -1.5) | -2.6 (-3.4, -1.8) | -2.6 (-3.5, -1.8) |
| **Educational attainment** |  |  |  |  |  |  |
| Higher tertiary or more |  | Ref. | Ref. |  | Ref. | Ref. |
| Lower tertiary |  | -0.7 (-1.3, -0.2) | -0.7 (-1.3, -0.2) |  | -0.2 (-0.8, 0.5) | -0.2 (-0.8, 0.5) |
| Upper secondary |  | -0.4 (-0.9, 0.2) | -0.3 (-0.8, 0.3) |  | -0.1 (-0.7, 0.6) | -0.1 (-0.7, 0.6) |
| Compulsory |  | 0.8 (0.2, 1.5) | 0.9 (0.2, 1.6) |  | -0.8 (-1.7, 0.1) | -0.8 (-1.8, 0.1) |
| **Economic activity** |  |  |  |  |  |  |
| Employed |  | Ref. | Ref. |  | Ref. | Ref. |
| Unemployed |  | 5.6 (5.2, 6.1) | 5.7 (5.3, 6.2) |  | 0.2 (0.0, 0.4) | 0.2 (-0.0, 0.4) |
| Students and pupils |  | 4.4 (3.9, 5.0) | 4.5 (4.0, 5.1) |  | 0.3 (-010, 0.6) | 0.3 (-0.1, 0.6) |
| Pensioners |  | 50.3 (48.6, 52.1) | 49.9 (48.1, 51.6) |  | 12.4 (11.8, 13.1) | 12.4 (11.8, 13.1) |
| Other economic activity |  | 2.7 (2.2, 3.1) | 3.3 (2.8, 3.7) |  | 0.5 (0.3, 0.8) | 0.5 (0.3, 0.8) |
| **No. of children < 18 years** |  |  |  |  |  |  |
| No children |  |  | 1.6 (1.2, 2.0) |  |  | 0.2 (-0.0, 0.4) |
| 1 child |  |  | Ref. |  |  | Ref. |
| 2 children |  |  | -1.2 (-1.5, -0.8) |  |  | 0.2 (0.1, 0.4) |
| 3 or more children |  |  | -2.0 (-2.5, -1.6) |  |  | 0.3 (0.1, 0.6) |
| **Constant** | 3.6 (3.2, 4.0) | 2.5 (1.9, 3.1) | 1.6 (1.0, 2.3) | 7.0 (6.7, 7.3) | 6.9 (6.2, 7.5) | 6.7 (6.0, 7.4) |

Notes*:* Coefficients from the OLS and FE models were multiplied by 100 to present percent changes in being prescribed psychotropic medication. Ref.: reference category. 95% confidence intervals in brackets. All analyses were controlled for 5-year age groups and year. Models were additionally controlled for educational attainment, economic activity and number of children in the family where mentioned. The ‘other economic activity’ included the categories other, unknown, conscripts, and conscientious objectors.

**Table E**―Predicted probabilities of being prescribed psychotropic medication for different living arrangements of men and women aged 25-39 years in 1995, for 1995 to 2007

|  | **Ordinary Least Squares** | | | **Individual Fixed Effects** | | |
| --- | --- | --- | --- | --- | --- | --- |
|  | **Model 1** | **Model 2** | **Model 3** | **Model 4** | **Model 5** | **Model 6** |
| The models controlled for: | Year, age | Year, age, education, economic activity | Year, age, education, economic activity, no. of children | Year, age | Year, age, education, economic activity | Year, age, education, economic activity, no. of children |
| **Men, Sample size (No. of obs.)** | 63077 (800650) | 63077 (800650) | 63077 (800650) | 63077 (800650) | 63077 (800650) | 63077 (800650) |
| **Living arrangement** |  |  |  |  |  |  |
| Married | 5.2 (5.1, 5.4) | 7.0 (6.9, 7.2) | 7.6 (7.4, 7.8) | 8.3 (8.2, 8.4) | 8.3 (8.2, 8.4) | 8.3 (8.2, 8.5) |
| Cohabiting | 6.4 (6.2, 6.7) | 7.3 (7.1, 7.6) | 7.3 (7.1, 7.6) | 7.9 (7.8, 8.1) | 7.9 (7.8, 8.1) | 7.9 (7.8, 8.1) |
| Living alone | 14.0 (13.6, 14.4) | 12.3 (11.9, 12.6) | 11.5 (11.1, 11.8) | 9.1 (9.0, 9.3) | 9.1 (8.9, 9.3) | 9.1 (8.9, 9.3) |
| Other | 13.4 (12.9, 14.0) | 9.3 (8.9, 9.8) | 8.5 (8.0, 8.9) | 8.5 (8.3, 8.7) | 8.5 (8.3, 8.7) | 8.5 (8.3, 8.7) |
| Unknown | 17.6 (16.3, 18.9) | 6.8 (5.5, 8.0) | 5.9 (4.6, 7.1) | 7.0 (6.6, 7.5) | 6.7 (6.3, 7.2) | 6.7 (6.3, 7.2) |
| All | 8.4 (8.2, 8.5) | 8.4 (8.3, 8.5) | 8.4 (8.3, 8.5) | 8.4 (8.4, 8.4) | 8.4 (8.4, 8.4) | 8.4 (8.4, 8.4) |
| **Women, Sample size (No. of obs.)** | 61101 (781368) | 61101 (781368) | 61101 (781368) | 61101 (781368) | 61101 (781368) | 61101 (781368) |
| **Living arrangement** |  |  |  |  |  |  |
| Married | 8.5 (8.4, 8.7) | 9.5 (9.4, 9.7) | 10.0 (9.8, 10.2) | 11.1 (10.9, 11.2) | 11.0 (10.9, 11.2) | 11.0 (10.9, 11.1) |
| Cohabiting | 10.4 (10.0, 10.7) | 10.6 (10.3, 10.9) | 10.3 (9.9, 10.6) | 10.8 (10.6, 11.0) | 10.8 (10.6, 11.0) | 10.8 (10.6, 11.0) |
| Living alone | 16.9 (16.5, 17.3) | 16.0 (15.6, 16.3) | 15.4 (15.0, 15.7) | 12.2 (12.0, 12.3) | 12.2 (12.0, 12.3) | 12.2 (12.0, 12.4) |
| Other | 18.6 (17.5, 19.7) | 11.6 (10.7, 12.5) | 10.0 (9.0, 10.9) | 11.8 (11.3, 12.2) | 11.8 (11.4, 12.2) | 11.8 (11.4, 12.2) |
| Unknown | 17.1 (14.9, 19.2) | 3.9 (2.0, 5.7) | 2.0 (0.1, 3.9) | 7.6 (6.7, 8.6) | 7.4 (6.5, 8.4) | 7.5 (6.5, 8.4) |
| All | 11.3 (11.1, 11.4) | 11.3 (11.1, 11.4) | 11.3 (11.1, 11.4) | 11.3 (11.2, 11.3) | 11.3 (11.2, 11.3) | 11.3 (11.2, 11.3) |

Notes: The probabilities were estimated by the margins command in Stata 14. 95% confidence intervals in brackets. All analyses were controlled for 5-year age groups and year. Models were additionally controlled for educational attainment, economic activity and number of children in the family where mentioned.

**Table F**―Percent differences in psychotropic medication for different living arrangements comparing parents with childless men and women aged 25-39 years in 1995, for 1995 to 2007

|  | **Ordinary Least Squares** | | **Individual Fixed Effects** | |
| --- | --- | --- | --- | --- |
|  | **Model 1** | **Model 2** | **Model 3** | **Model 4** |
| The model controlled for: | Year, age | Year, age, education, economic activity | Year, age | Year, age, education, economic activity |
| **Men** |  |  |  |  |
| **Childless men, Sample size (No. of obs.)** | 46350 (381956) | 46350 (381956) | 46350 (381956) | 46350 (381956) |
| **Living arrangement** |  |  |  |  |
| Married | -0.7 (-1.3, -0.1) | 0.4 (-0.2, 0.9) | 0.1 (-0.3, 0.5) | 0.2 (-0.2, 0.6) |
| Cohabiting | Ref. | Ref. | Ref. | Ref. |
| Living alone | 6.7 (6.1, 7.2) | 4.2 (3.8, 4.7) | 1.4 (1.1, 1.7) | 1.3 (1.0, 1.6) |
| Other | 5.9 (5.3, 6.6) | 0.9 (0.3, 1.5) | 0.9 (0.6, 1.3) | 0.9 (0.5, 1.2) |
| Unknown | 9.8 (8.5, 11.2) | -2.3 (-3.6, -1.0) | -0.8 (-1.4, -0.3) | -1.1 (-1.7, -0.6) |
| **Fathers, Sample size (No. of obs.)** | 44492 (418694) | 44492 (418694) | 44492 (418694) | 44492 (418694) |
| **Living arrangement** |  |  |  |  |
| Married | -0.5 (-0.9, -0.2) | -0.2 (-0.5, 0.2) | 0.4 (0.1, 0.7) | 0.4 (0.1, 0.7) |
| Cohabiting | Ref. | Ref. | Ref. | Ref. |
| Living alone | 2.8 (1.8, 3.8) | 2.0 (1.1, 3.0) | 0.5 (0.0, 1.0) | 0.4 (-0.1, 0.9) |
| Other | 4.5 (2.0, 7.0) | 2.2 (-0.3, 4.6) | 0.2 (-1.5, 1.9) | 0.3 (-1.4, 2.0) |
| Unknown | NA | NA | NA | NA |
| **Comparing parenthood status by living arrangement – p value** |  |  |  |  |
| **Living arrangement** |  |  |  |  |
| Married | 0.618 | 0.095 | 0.188 | 0.361 |
| Cohabiting | NA | NA | NA | NA |
| Living alone | <0.001 | <0.001 | 0.007 | 0.005 |
| Other | 0.267 | 0.321 | 0.463 | 0.542 |
| Unknown | NA | NA | NA | NA |
| **Women** |  |  |  |  |
| **Childless women, Sample size (No. of obs.)** | 35534 (255638) | 35534 (255638) | 35534 (255638) | 35534 (255638) |
| **Living arrangement** |  |  |  |  |
| Married | -1.7 (-2.5, -1.0) | -0.7 (-1.4, 0.0) | 0.0 (-0.5, 0.5) | 0.1 (-0.4, 0.6) |
| Cohabiting | Ref. | Ref. | Ref. | Ref. |
| Living alone | 6.6 (5.8, 7.3) | 5.1 (4.4, 5.8) | 1.4 (1.0, 1.8) | 1.4 (1.0, 1.8) |
| Other | 6.5 (5.2, 7.7) | -1.2 (-2.2, 0.2) | 1.1 (0.5, 1.6) | 0.9 (0.4, 1.5) |
| Unknown | 4.4 (2.2, 6.6) | -10.6 (-12.7, -8.4) | -3.2 (-4.2, 2.2) | -3.7 (-4.7, -2.7) |
| **Mothers, Sample size (No. of obs.)** | 49431 (525730) | 49431 (525730) | 49431 (525730) | 49431 (525730) |
| **Living arrangement** |  |  |  |  |
| Married | -0.8 (-1.2, -0.3) | -0.4 (-0.8, 0.0) | 0.3 (-0.0, 0.7) | 0.3 (-0.0, 0.7) |
| Cohabiting | Ref. | Ref. | Ref. | Ref. |
| Living alone | 5.7 (5.1, 6.3) | 5.1 (4.5, 5.7) | 1.4 (1.1, 1.8) | 1.4 (1.1, 1.8) |
| Other | 5.9 (-0.8, 12.7) | 0.7 (-4.6, 6.0) | 3.6 (0.2, 7.1) | 3.6 (0.7, 7.1) |
| Unknown | NA | NA | NA | NA |
| **Comparing parenthood status by living arrangement – p value** |  |  |  |  |
| **Living arrangement** |  |  |  |  |
| Married | 0.031 | 0.540 | 0.372 | 0.415 |
| Cohabiting | NA | NA | NA | NA |
| Living alone | 0.081 | 0.934 | 0.943 | 0.835 |
| Other | 0.880 | 0.492 | 0.161 | 0.157 |
| Unknown | NA | NA | NA | NA |

Notes: Coefficients from the OLS and FE models were multiplied by 100 to present percent changes in being prescribed psychotropic medication. Ref.: reference category. 95% confidence intervals in brackets. All analyses were controlled for 5-year age groups and year. Models were additionally controlled for educational attainment and economic activity where mentioned. Men and women were defined as fathers and mothers respectively, when they had at least one child under the age of 18 years living in their family. NA: Not available, as for individuals in the unknown category, information on children was not available.

**Table G**―Predicted probabilities of psychotropic medication for different living arrangements comparing parents with childless men and women aged 25-39 years in 1995, for 1995 to 2007

|  | **Ordinary Least Squares** | | **Individual Fixed Effects** | |
| --- | --- | --- | --- | --- |
|  | **Model 1** | **Model 2** | **Model 3** | **Model 4** |
| The model controlled for: | Year, age | Year, age, education,  economic activity | Year, age | Year, age, education, economic activity |
| **Men** |  |  |  |  |
| **Childless men, Sample size (No. of obs.)** | 46350 (381956) | 46350 (381956) | 46350 (381956) | 46350 (381956) |
| **Living arrangement** |  |  |  |  |
| Married | 6.8 (6.4, 7.3) | 10.4 (9.9, 10.8) | 11.2 (10.9, 11.5) | 11.3 (11.0, 11.6) |
| Cohabiting | 7.5 (7.1, 7.9) | 10.0 (9.6, 10.4) | 11.1 (10.9, 11.3) | 11.1 (10.9, 11.4) |
| Living alone | 14.2 (13.8, 14.6) | 14.2 (13.9, 14.6) | 12.5 (12.3, 12.6) | 12.5 (12.3, 12.6) |
| Other | 13.4 (12.9, 14.0) | 10.9 (10.4, 11.4) | 12.0 (11.8, 12.2) | 12.0 (11.8, 12.2) |
| Unknown | 17.3 (16.0, 18.7) | 7.7 (6.4, 8.9) | 10.3 (9.8, 10.8) | 10.0 (9.5, 10.5) |
| All | 11.9 (11.6, 12.1) | 11.8 (11.6, 12.1) | 11.8 (11.8, 11.9) | 11.8 (11.8, 11.9) |
| **Fathers, Sample size (No. of obs.)** | 44492 (418694) | 44492 (418694) | 44492 (418694) | 44492 (418694) |
| **Living arrangement** |  |  |  |  |
| Married | 5.0 (4.8, 5.2) | 5.1 (5.0, 5.3) | 5.3 (5.3, 5.4) | 5.3 (5.2, 5.4) |
| Cohabiting | 5.6 (5.2, 5.9) | 5.3 (5.0, 5.6) | 4.9 (4.7, 5.2) | 4.9 (4.7, 5.2) |
| Living alone | 8.3 (7.4, 9.3) | 7.4 (6.5, 8.3) | 5.4 (5.0, 5.9) | 5.4 (4.9, 5.8) |
| Other | 10.0 (7.5, 12.5) | 7.5 (5.1, 9.9) | 5.1 (3.4, 6.8) | 5.2 (3.5, 6.9) |
| Unknown | NA | NA | NA | NA |
| All | 5.2 (5.1, 5.4) | 5.2 (5.1, 5.4) | 5.2 (5.2, 5.3) | 5.2 (5.2, 5.3) |
| **Women** |  |  |  |  |
| **Childless women, Sample size (No. of obs.)** | 35534 (255638) | 35534 (255638) | 35534 (255638) | 35534 (255638) |
| **Living arrangement** |  |  |  |  |
| Married | 11.0 (10.5, 11.6) | 13.6 (13.1, 14.1) | 15.0 (14.7, 15.4) | 15.1 (14.7, 15.4) |
| Cohabiting | 12.8 (12.2, 13.3) | 14.2 (13.7, 14.8) | 15.0 (14.7, 15.3) | 15.0 (14.7, 15.3) |
| Living alone | 19.3 (18.7, 19.9) | 19.3 (18.8, 19.8) | 16.4 (16.2, 16.6) | 16.4 (16.1, 16.6) |
| Other | 19.2 (18.1, 20.3) | 13.0 (12.1, 14.0) | 16.0 (15.6, 16.5) | 16.0 (15.5, 16.4) |
| Unknown | 17.1 (15.0, 19.3) | 3.7 (1.6, 5.7) | 11.8 (10.8, 12.7) | 11.3 (10.3, 12.3) |
| All | 15.5 (15.2, 15.9) | 15.5 (15.2, 15.9) | 15.5 (15.5, 15.6) | 15.5 (15.5, 15.6) |
| **Mothers, Sample size (No. of obs.)** | 49431 (525730) | 49431 (525730) | 49431 (525730) | 49431 (525730) |
| **Living arrangement** |  |  |  |  |
| Married | 8.0 (7.8, 8.2) | 8.2 (8.0, 8.4) | 9.0 (8.9, 9.1) | 9.0 (8.9, 9.1) |
| Cohabiting | 8.7 (8.3, 9.1) | 8.6 (8.2, 9.0) | 8.7 (8.5, 9.0) | 8.7 (8.5, 9.0) |
| Living alone | 14.4 (13.9, 14.9) | 13.7 (13.3, 14.2) | 10.2 (9.9, 10.4) | 10.2 (9.9, 10.4) |
| Other | 14.7 (7.9, 21.4) | 9.3 (4.0, 14.5) | 12.4 (8.9, 15.9) | 12.3 (8.8, 15.8) |
| Unknown | NA | NA | NA | NA |
| All | 9.2 (9.0, 9.4) | 9.2 (9.0, 9.4) | 9.2 (9.1, 9.2) | 9.2 (9.1, 9.2) |

Notes: The probabilities were estimated by the margins command in Stata 14. 95% confidence intervals in brackets. All analyses were controlled for 5-year age groups and year. Models were additionally controlled for educational attainment and economic activity where mentioned. Men and women were defined as fathers and mothers respectively, when they had at least one child under the age of 18 years living in their family. NA: Not available, as for individuals in the unknown category, information on children was not available.

**Table H**―Percent differences in psychotropic medication by subcategory for different living arrangements of men and women aged 25-39 years in 1995, for 1995 to 2007

|  | **Ordinary Least Squares** | | | **Individual Fixed Effects** | | |
| --- | --- | --- | --- | --- | --- | --- |
|  | **Model 1** | **Model 2** | **Model 3** | **Model 4** | **Model 5** | **Model 6** |
| The models controlled for: | Year, age | Year, age, education, economic activity | Year, age, education, economic activity, no. of children | Year, age | Year, age, education, economic activity | Year, age, education, economic activity, no. of children |
| **Men, Sample size (No. of obs.)** | 63077 (800650) | 63077 (800650 | 63077 (800650 | 63077 (800650 | 63077 (800650 | 63077 (800650 |
| **All psychotropic medication, living arrangement** | |  |  |  |  |  |
| Married | -1.2 (-1.5, -0.9) | -0.3 (-0.6, -0.0) | 0.3 (-0.0, 0.6) | 0.1 (-0.1, 0.3) | 0.1 (-0.1, 0.3) | 0.3 (0.1, 0.5) |
| Cohabiting | Ref. | Ref. | Ref. | Ref. | Ref. | Ref. |
| Living alone | 7.6 (7.1, 8.0) | 4.9 (4.5, 5.3) | 4.2 (3.7, 4.6) | 1.5 (1.3, 1.7) | 1.5 (1.3, 1.6) | 1.2 (1.0, 1.4) |
| Other | 7.0 (6.4, 7.6) | 2.0 (1.5, 2.5) | 1.1 (0.6, 1.7) | 0.8 (0.6, 1.0) | 0.7 (0.5, 1.0) | 0.5 (0.2, 0.7) |
| Unknown | 11.2 (9.8, 12.5) | -0.6 (-1.8, 0.7) | -1.4 (-2.7, 0.1) | -0.7 (-1.1, -0.3) | -1.1 (-1.5, -0.6) | -1.4 (-1.8, -0.9) |
| **Antidepressants, living arrangement** |  |  |  |  |  |  |
| Married | -0.5 (-0.7, -0.2) | -0.0 (-0.3, 0.2) | 0.2 (-0.0, 0.4) | 0.2 (-0.0, 0.3) | 0.2 (0.0, 0.4) | 0.2 (0.1, 0.4) |
| Cohabiting | Ref. | Ref. | Ref. | Ref. | Ref. | Ref. |
| Living alone | 4.1 (3.8, 4.4) | 2.9 (2.6, 3.2) | 2.6 (2.3, 2.9) | 1.1 (0.9, 1.2) | 1.0 (0.8, 1.2) | 0.9 (0.8, 1.1) |
| Other | 2.5 (2.1, 2.8) | 0.2 (-0.1, 0.6) | -0.1 (-0.5, 0.3) | 0.5 (0.3, 0.7) | 0.4 (0.3, 0.6) | 0.4 (0.1, 0.6) |
| Unknown | 3.7 (2.9, 4.5) | -1.5 (-2.3, -0.7) | -1.8 (-2.7, -1.0) | 0.2 (-0.2, 0.6) | -0.1 (-0.5, 0.3) | -0.2 (-0.6, 0.2) |
| **Antipsychotics, living arrangement** |  |  |  |  |  |  |
| Married | -0.4 (-0.5, -0.3) | 0.0 (-0.1, 0.2) | 0.2 (0.1, 0.3) | -0.1 (-0.2, -0.0) | -0.1 (-0.2, 0.0) | -0.0 (-0.1, 0.1) |
| Cohabiting | Ref. | Ref. | Ref. | Ref. | Ref. | Ref. |
| Living alone | 3.6 (3.3, 3.9) | 2.0 (1.8, 2.2) | 1.7 (1.5, 2.0) | 0.5 (0.4, 0.6) | 0.5 (0.4, 0.5) | 0.4 (0.3, 0.5) |
| Other | 5.0 (4.5, 54) | 1.9 (1.6, 2.2) | 1.6 (1.2, 1.9) | 0.2 (0.1, 0.3) | 0.1 (0.0, 0.3) | 0.1 (-0.1, 0.2) |
| Unknown | 8.4 (7.2, 9.5) | 0.9 (-0.1, 1.9) | 0.6 (-0.4, 1.6) | -0.0 (-0.2, 0.2) | -0.3 (-0.5, -0.1) | -0.3 (-0.5, -0.1) |
| **Antimanic agents, living arrangement** |  |  |  |  |  |  |
| Married | -0.3 (-0.4, -0.1) | -0.1 (-0.2, 0.1) | 0.1 (-0.1, 0.2) | -0.1 (-0.2, -0.1) | -0.1 (-0.2, -0.0) | -0.0 (-0.1, 0.0) |
| Cohabiting | Ref. | Ref. | Ref. | Ref. | Ref. | Ref. |
| Living alone | 1.4 (1.2, 1.6) | 0.6 (0.4, 0.8) | 0.4 (0.2, 0.6) | 0.3 (0.2, 0.4) | 0.3 (0.2, 0.3) | 0.2 (0.1, 0.3) |
| Other | 2.2 (1.8, 2.5) | 0.7 (0.5, 1.0) | 0.5 (0.2, 0.8) | 0.0 (-0.1, 0.1) | -0.0 (-0.1, 0.1) | -0.1 (-0.2, -0.0) |
| Unknown | 3.4 (2.7, 4.1) | -0.0 (-0.7, 0.7) | -0.2 (-0.9, 0.5) | 0.1 (-0.0, 0.3) | -0.0 (-0.2, 0.1) | -0.1 (-0.3, 0.1) |
| **ASH medication, living arrangement** |  |  |  |  |  |  |
| Married | -0.8 (-1.0, -0.6) | -0.2 (-0.4, -0.0) | 0.2 (-0.0, 0.4) | 0.1 (-0.0, 0.3) | 0.1 (-0.0, 0.3) | 0.3 (0.1, 0.4) |
| Cohabiting | Ref. | Ref. | Ref. | Ref. | Ref. | Ref. |
| Living alone | 4.3 (4.0, 4.7) | 3.0 (2.7, 3.2) | 2.4 (2.1, 2.7) | 1.0 (0.8, 1.1) | 0.9 (0.8, 1.1) | 0.8 (0.6, 0.9) |
| Other | 3.0 (2.6, 3.4) | 0.3 (-0.0, 0.7) | -0.3 (-0.6, 0.1) | 0.5 (0.3, 0.7) | 0.5 (0.3, 0.7) | 0.3 (0.1, 0.5) |
| Unknown | 6.8 (5.9, 7.7) | 0.6 (-0.3, 1.5) | -0.0 (-1.0, 0.9) | 0.0 (-0.3, 0.4) | -0.1 (-0.5, 0.2) | -0.3 (-0.7, 0.0) |
| **Women, Sample size (No. of obs.)** | 61101 (781368) | 61101 (781368) | 61101 (781368) | 61101 (781368) | 61101 (781368) | 61101 (781368) |
| **All psychotropic medication, living arrangement** | |  |  |  |  |  |
| Married | -1.8 (-2.2, -1.4) | -1.1 (-1.5, -0.7) | -0.2 (-0.6, 0.2) | 0.1 (-0.2, 0.3) | 0.1 (-0.2, 0.3) | 0.1 (-0.2, 0.3) |
| Cohabiting | Ref. | Ref. | Ref. | Ref. | Ref. | Ref. |
| Living alone | 6.6 (6.1, 7.1) | 5.4 (4.9, 5.8) | 5.1 (4.7, 5.6) | 1.4 (1.2, 1.6) | 1.4 (1.1, 1.6) | 1.4 (1.1, 1.6) |
| Other | 8.2 (7.1, 9.4) | 1.0 (0.0, 1.9) | -0.3 (-1.3, 0.7) | 1.1 (0.6, 1.5) | 1.1 (0.6, 1.5) | 1.0 (0.6, 1.5) |
| Unknown | 6.7 (4.6, 8.9) | -6.8 (-8.7, -4.9) | -8.3 (-10.2, -6.3) | -2.3 (-3.2, -1.5) | -2.6 (-3.4, -1.8) | -2.6 (-3.5, -1.8) |
| **Antidepressants, living arrangement** |  |  |  |  |  |  |
| Married | -1.0 (-1.3, 0.7) | -0.6 (-0.9, -0.3) | -0.2 (-0.5, 0.1) | 0.1 (-0.2, 0.3) | 0.1 (-0.2, 0.3) | -0.0 (-0.3, 0.2) |
| Cohabiting | Ref. | Ref. | Ref. | Ref. | Ref. | Ref. |
| Living alone | 4.4 (4.0, 4.8) | 3.8 (3.4, 4.2) | 3.7 (3.3, 4.1) | 1.0 (0.8, 1.2) | 1.0 (0.8, 1.2) | 1.0 (0.8, 1.2) |
| Other | 2.1 (1.4, 2.8) | -1.5 (-2.2, -0.8) | -2.0 (-2.7, -1.3) | 0.9 (0.5, 1.2) | 0.8 (0.4, 1.2) | 0.9 (0.5, 1.3) |
| Unknown | 1.3 (-0.0, 2.6) | -5.5 (-6.9, -4.2) | -6.1 (-7.5, -4.7) | -0.5 (-1.2, 0.3) | -0.7 (-1.5, -0.0) | -0.6 (-1.3, 0.1) |
| **Antipsychotics, living arrangement** |  |  |  |  |  |  |
| Married | -0.6 (-0.8, -0.5) | -0.2 (-0.4, -0.1) | 0.2 (0.0, 0.3) | -0.0 (-0.1, 0.0) | -0.0 (-0.1, 0.0) | 0.0 (-0.1, 0.1) |
| Cohabiting | Ref. | Ref. | Ref. | Ref. | Ref. | Ref. |
| Living alone | 2.3 (2.0, 2.6) | 1.5 (1.3, 1.7) | 1.4 (1.2, 1.6) | 0.3 (0.2, 0.4) | 0.3 (0.2, 0.4) | 0.3 (0.2, 0.4) |
| Other | 6.7 (5.8, 7.6) | 1.6 (0.9, 2.3) | 0.9 (0.1, 1.6) | 0.2 (-0.0, 0.3) | 0.1 (-0.0, 0.3) | 0.1 (-0.1, 0.3) |
| Unknown | 7.4 (5.6, 9.1) | -2.2 (-3.7, -0.6) | -3.0 (-4.5, -1.4) | -0.7 (-1.0, -0.3) | -0.9 (-1.2, -0.6) | -1.0 (-1.3, -0.7) |
| **Antimanic agents, living arrangement** |  |  |  |  |  |  |
| Married | -0.3 (-0.5, -0.2) | -0.1 (-0.3, 0.0) | 0.1 (-0.1, 0.2) | 0.0 (-0.1, 0.1) | 0.0 (-0.1, 0.1) | 0.0 (-0.1, 0.1) |
| Cohabiting | Ref. | Ref. | Ref. | Ref. | Ref. | Ref. |
| Living alone | 1.0 (0.8, 1.2) | 0.6 (0.4, 0.8) | 0.5 (0.4, 0.7) | 0.3 (0.2, 0.3) | 0.2 (0.2, 0.3) | 0.2 (0.2, 0.3) |
| Other | 3.5 (2.9, 4.2) | 0.9 (0.4, 1.5) | 0.6 (0.1, 1.2) | 0.4 (0.3, 0.5) | 0.4 (0.2, 0.5) | 0.4 (0.2, 0.5) |
| Unknown | 4.6 (3.2, 6.0) | -0.2 (-1.5, 1.1) | -0.5 (-1.8, 0.7) | 0.2 (-0.0, 0.5) | 0.1 (-0.2, 0.3) | 0.0 (-0.2, 0.3) |
| **ASH medication, living arrangement** |  |  |  |  |  |  |
| Married | -1.1 (-1.3, -0.8) | -0.7 (-0.9, -0.4) | -0.0 (-0.3, 0.2) | 0.2 (-0.0, 0.3) | 0.2 (-0.0, 0.4) | 0.2 (0.0, 0.4) |
| Cohabiting | Ref. | Ref. | Ref. | Ref. | Ref. | Ref. |
| Living alone | 3.7 (3.4, 4.1) | 3.1 (2.8, 3.4) | 2.9 (2.6, 3.2) | 0.6 (0.4, 0.8) | 0.6 (0.4, 0.8) | 0.6 (0.4, 0.8) |
| Other | 3.9 (3.2, 4.6) | 0.2 (-0.5, 0.8) | -0.8 (-1.4, -0.1) | 0.6 (0.3, 0.9) | 0.6 (0.3, 1.0) | 0.5 (0.2, 0.9) |
| Unknown | 3.9 (2.5, 5.3) | -3.0 (-4.4, -1.7) | -4.1 (-5.5, -2.7) | -0.5 (-1.1, 0.1) | -0.6 (-1.3, 0.0) | -0.8 (-1.5, -0.2) |

Notes: Coefficients from the OLS and FE models were multiplied by 100 to present percent changes in being prescribed psychotropic medication. Ref.: reference category. 95% confidence intervals in brackets. ASH medication: anxiolytic/sedative/hypnotic medication. Anatomical Therapeutic Chemical (ATC) codes available in Table A. All analyses were controlled for 5-year age groups and year. Models were additionally controlled for educational attainment, economic activity and number of children where mentioned.

**Table I**―Predicted probabilities of psychotropic medication by subcategory for different living arrangements of men and women aged 25-39 years in 1995, for 1995 to 2007

|  | **Ordinary Least Squares** | | | **Individual Fixed Effects** | | |
| --- | --- | --- | --- | --- | --- | --- |
|  | **Model 1** | **Model 2** | **Model 3** | **Model 4** | **Model 5** | **Model 6** |
| The models controlled for: | Year, age | Year, age, education, economic activity | Year, age, education, economic activity, no. of children | Year, age | Year, age, education, economic activity | Year, age, education, economic activity, no. of children |
| **Men, Sample size (No. of obs.)** | 63077 (800650) | 63077 (800650) | 63077 (800650) | 63077 (800650) | 63077 (800650) | 63077 (800650) |
| **All psychotropic medication, living arrangement** | |  |  |  |  |  |
| Married | 5.2 (5.1, 5.4) | 7.0 (6.9, 7.2) | 7.6 (7.4, 7.8) | 8.3 (8.2, 8.4) | 8.3 (8.2, 8.4) | 8.3 (8.2, 8.5) |
| Cohabiting | 6.4 (6.2, 6.7) | 7.3 (7.1, 7.6) | 7.3 (7.1, 7.6) | 7.9 (7.8, 8.1) | 7.9 (7.8, 8.1) | 7.9 (7.8, 8.1) |
| Living alone | 14.0 (13.6, 14.4) | 12.3 (11.9, 12.6) | 11.5 (11.1, 11.8) | 9.1 (9.0, 9.3) | 9.1 (8.9, 9.3) | 9.1 (8.9, 9.3) |
| Other | 13.4 (12.9, 14.0) | 9.3 (8.9, 9.8) | 8.5 (8.0, 8.9) | 8.5 (8.3, 8.7) | 8.5 (8.3, 8.7) | 8.5 (8.3, 8.7) |
| Unknown | 17.6 (16.3, 18.9) | 6.8 (5.5, 8.0) | 5.9 (4.6, 7.1) | 7.0 (6.6, 7.5) | 6.7 (6.3, 7.2) | 6.7 (6.3, 7.2) |
| All | 8.4 (8.2, 8.5) | 8.4 (8.3, 8.5) | 8.4 (8.3, 8.5) | 8.4 (8.4, 8.4) | 8.4 (8.4, 8.4) | 8.4 (8.4, 8.4) |
| **Antidepressants, living arrangement** |  |  |  |  |  |  |
| Married | 3.2 (3.1, 3.3) | 4.0 (3.9, 4.2) | 4.3 (4.1, 4.4) | 4.6 (4.5, 4.7) | 4.6 (4.5, 4.7) | 4.6 (4.5, 4.7) |
| Cohabiting | 3.7 (3.5, 3.9) | 4.1 (3.9, 4.3) | 4.1 (3.9, 4.3) | 4.2 (4.1, 4.4) | 4.2 (4.1, 4.4) | 4.2 (4.1, 4.4) |
| Living alone | 7.8 (7.5, 8.1) | 7.0 (6.7, 7.3) | 6.7 (6.4, 7.0) | 5.2 (5.0, 5.3) | 5.2 (5.0, 5.3) | 5.2 (5.0, 5.3) |
| Other | 6.2 (5.8, 6.5) | 4.3 (4.0, 4.6) | 4.0 (3.6, 4.3) | 4.7 (4.5, 4.9) | 4.7 (4.5, 4.9) | 4.7 (4.5, 4.9) |
| Unknown | 7.4 (6.7, 8.2) | 2.6 (1.8, 3.4) | 2.3 (1.5, 3.1) | 4.7 (4.3, 5.1) | 4.5 (4.1, 4.9) | 4.5 (4.1, 4.9) |
| All | 4.7 (4.5, 4.8) | 4.7 (4.6, 4.8) | 4.7 (4.6, 4.8) | 4.7 (4.6, 4.7) | 4.7 (4.6, 4.7) | 4.7 (4.6, 4.7) |
| **Antipsychotics, living arrangement** |  |  |  |  |  |  |
| Married | 0.6 (0.5, 0.7) | 1.6 (1.6, 1.7) | 1.8 (1.7, 1.9) | 2.2 (2.1, 2.2) | 2.2 (2.1, 2.2) | 2.2 (2.1, 2.2) |
| Cohabiting | 1.0 (0.9, 1.1) | 1.6 (1.5, 1.7) | 1.6 (1.5, 1.7) | 2.2 (2.1, 2.2) | 2.2 (2.1, 2.3) | 2.2 (2.1, 2.3) |
| Living alone | 4.6 (4.3, 4.8) | 3.6 (3.4, 3.8) | 3.3 (3.1, 3.5) | 2.6 (2.5, 2.7) | 2.6 (2.5, 2.6) | 2.6 (2.5, 2.6) |
| Other | 5.9 (5.5, 6.4) | 3.5 (3.2, 3.8) | 3.2 (2.9, 3.5) | 2.2 (2.1, 2.4) | 2.2 (2.1, 2.3) | 2.2 (2.1, 2.3) |
| Unknown | 9.3 (8.2, 10.5) | 2.5 (1.5, 3.5) | 2.2 (1.2, 3.2) | 1.9 (1.7, 2.1) | 1.7 (1.5, 1.9) | 1.7 (1.5, 1.9) |
| All | 2.3 (2.2, 2.3) | 2.3 (2.2, 2.3) | 2.3 (2.2, 2.3) | 2.3 (2.2, 2.3) | 2.3 (2.2, 2.3) | 2.3 (2.2, 2.3) |
| **Antimanic agents, living arrangement** |  |  |  |  |  |  |
| Married | 0.6 (0.5, 0.7) | 1.1 (1.0, 1.1) | 1.2 (1.1, 1.3) | 1.3 (1.2, 1.3) | 1.3 (1.2, 1.3) | 1.3 (1.2, 1.3) |
| Cohabiting | 0.9 (0.8, 1.0) | 1.2 (1.0, 1.3) | 1.2 (1.1, 1.3) | 1.4 (1.3, 1.4) | 1.4 (1.3, 1.4) | 1.3 (1.3, 1.4) |
| Living alone | 2.2 (2.1, 2.4) | 1.8 (1.6, 1.9) | 1.6 (1.4, 1.8) | 1.6 (1.5, 1.6) | 1.5 (1.5, 1.6) | 1.5 (1.5, 1.6) |
| Other | 3.0 (2.7, 3.3) | 1.9 (1.6, 2.1) | 1.7 (1.4, 1.9) | 1.2 (1.1, 1.3) | 1.2 (1.1, 1.3) | 1.2 (1.1, 1.3) |
| Unknown | 4.3 (3.5, 5.0) | 1.1 (0.5, 1.8) | 0.9 (0.2, 1.6) | 1.3 (1.1, 1.5) | 1.2 (1.0, 1.3) | 1.2 (1.0, 1.3) |
| All | 1.3 (1.3, 1.4) | 1.3 (1.3, 1.4) | 1.3 (1.3, 1.4) | 1.3 (1.3, 1.4) | 1.3 (1.3, 1.4) | 1.3 (1.3, 1.4) |
| **ASH medication, living arrangement** |  |  |  |  |  |  |
| Married | 2.7 (2.7, 2.9) | 3.8 (3.6, 3.9) | 4.2 (4.0, 4.3) | 4.5 (4.4, 4.6) | 4.5 (4.4, 4.6) | 4.5 (4.4, 4.6) |
| Cohabiting | 3.5 (3.4, 3.7) | 4.0 (3.8, 4.2) | 4.0 (3.8, 4.2) | 4.3 (4.1, 4.4) | 4.3 (4.1, 4.4) | 4.3 (4.1, 4.4) |
| Living alone | 7.9 (7.6, 8.2) | 6.9 (6.7, 7.2) | 6.4 (6.1, 6.6) | 4.9 (4.8, 5.0) | 4.9 (4.8, 5.0) | 4.9 (4.8, 5.0) |
| Other | 6.5 (6.2, 6.9) | 4.3 (4.0, 4.7) | 3.7 (3.4, 4.1) | 4.5 (4.3, 4.7) | 4.5 (4.3, 4.7) | 4.5 (4.3, 4.7) |
| Unknown | 10.3 (9.4, 11.3) | 4.6 (3.7, 5.5) | 4.0 (3.1, 4.9) | 4.2 (3.8, 4.5) | 4.0 (3.7, 4.4) | 4.0 (3.7, 4.4) |
| All | 4.5 (4.4, 4.6) | 4.5 (4.4, 4.6) | 4.5 (4.4, 4.6) | 4.5 (4.5, 4.6) | 4.5 (4.5, 4.6) | 4.5 (4.5, 4.6) |
| **Women, Sample size (No. of obs.)** | 61101 (781368) | 61101 (781368) | 61101 (781368) | 61101 (781368) | 61101 (781368) | 61101 (781368) |
| **All psychotropic medication, living arrangement** | |  |  |  |  |  |
| Married | 8.5 (8.4, 8.7) | 9.5 (9.4, 9.7) | 10.0 (9.8, 10.2) | 11.1 (10.9, 11.2) | 11.0 (10.9, 11.2) | 11.0 (10.9, 11.1) |
| Cohabiting | 10.4 (10.0, 10.7) | 10.6 (10.3, 10.9) | 10.3 (9.9, 10.6) | 10.8 (10.6, 11.0) | 10.8 (10.6, 11.0) | 10.8 (10.6, 11.0) |
| Living alone | 16.9 (16.5, 17.3) | 16.0 (15.6, 16.3) | 15.4 (15.0, 15.7) | 12.2 (12.0, 12.3) | 12.2 (12.0, 12.3) | 12.2 (12.0, 12.4) |
| Other | 18.6 (17.5, 19.7) | 11.6 (10.7, 12.5) | 10.0 (9.0, 10.9) | 11.8 (11.3, 12.2) | 11.8 (11.4, 12.2) | 11.8 (11.4, 12.2) |
| Unknown | 17.1 (14.9, 19.2) | 3.9 (2.0, 5.7) | 2.0 (0.1, 3.9) | 7.6 (6.7, 8.6) | 7.4 (6.5, 8.4) | 7.5 (6.5, 8.4) |
| All | 11.3 (11.1, 11.4) | 11.3 (11.1, 11.4) | 11.3 (11.1, 11.4) | 11.3 (11.2, 11.3) | 11.3 (11.2, 11.3) | 11.3 (11.2, 11.3) |
| **Antidepressants, living arrangement** |  |  |  |  |  |  |
| Married | 5.8 (5.7, 6.0) | 6.3 (6.2, 6.5) | 6.5 (6.4, 6.7) | 7.2 (7.1, 7.3) | 7.2 (7.1, 7.3) | 7.2 (7.1, 7.3) |
| Cohabiting | 6.8 (6.5, 7.1) | 6.9 (6.7, 7.2) | 6.8 (6.5, 7.0) | 7.0 (6.8, 7.2) | 7.0 (6.8, 7.2) | 7.0 (6.8, 7.2) |
| Living alone | 11.2 (10.9, 11.5) | 10.7 (10.4, 11.0) | 10.4 (10.1, 10.7) | 8.1 (7.9, 8.2) | 8.1 (7.9, 8.2) | 8.1 (7.9, 8.3) |
| Other | 8.9 (8.3, 9.6) | 5.4 (4.8, 6.1) | 4.8 (4.1, 5.4) | 7.8 (7.4, 8.2) | 7.8 (7.4, 8.2) | 7.9 (7.5, 8.2) |
| Unknown | 8.1 (6.8, 9.4) | 1.4 (0.1, 2.7) | 0.6 (-0.7, 2.0) | 6.3 (5.4, 7.1) | 6.0 (5.2, 6.9) | 6.1 (5.2, 6.9) |
| All | 7.4 (7.2, 7.5) | 7.4 (7.3, 7.5) | 7.4 (7.3, 7.5) | 7.4 (7.3, 7.4) | 7.4 (7.3, 7.4) | 7.4 (7.3, 7.4) |
| **Antipsychotics, living arrangement** |  |  |  |  |  |  |
| Married | 1.0 (0.9, 1.0) | 1.6 (1.5, 1.7) | 1.8 (1.7, 1.9) | 2.1 (2.0, 2.1) | 2.1 (2.0, 2.1) | 2.1 (2.0, 2.1) |
| Cohabiting | 1.6 (1.4, 1.7) | 1.8 (1.7, 2.0) | 1.7 (1.5, 1.8) | 2.0 (1.9, 2.1) | 2.0 (1.9, 2.1) | 2.0 (1.9, 2.1) |
| Living alone | 3.9 (3.7, 4.1) | 3.3 (3.1, 3.5) | 3.0 (2.9, 3.2) | 2.3 (2.2, 2.3) | 2.3 (2.2, 2.3) | 2.3 (2.2, 2.3) |
| Other | 8.3 (7.4, 9.2) | 3.4 (2.7, 4.1) | 2.5 (1.8, 3.2) | 2.1 (1.9, 2.2) | 2.1 (1.9, 2.2) | 2.1 (1.9, 2.2) |
| Unknown | 9.0 (7.2, 10.7) | -0.3 (-1.9, 1.2) | -1.3 (-2.9, 0.2) | 0.8 (0.5, 1.2) | 0.6 (0.3, 1.0) | 0.6 (0.3, 1.0) |
| All | 2.1 (2.0, 2.2) | 2.1 (2.0, 2.2) | 2.1 (2.0, 2.2) | 2.1 (2.1, 2.1) | 2.1 (2.1, 2.1) | 2.1 (2.1, 2.1) |
| **Antimanic agents, living arrangement** |  |  |  |  |  |  |
| Married | 0.8 (0.7, 0.9) | 1.1 (1.0, 1.2) | 1.2 (1.1, 1.3) | 1.3 (1.3, 1.4) | 1.3 (1.3, 1.4) | 1.3 (1.3, 1.4) |
| Cohabiting | 1.1 (1.0, 1.3) | 1.2 (1.1, 1.4) | 1.2 (1.0, 1.3) | 1.2 (1.2, 1.3) | 1.2 (1.2, 1.3) | 1.2 (1.2, 1.3) |
| Living alone | 2.1 (2.0, 2.3) | 1.8 (1.7, 2.0) | 1.7 (1.6, 1.8) | 1.4 (1.4, 1.5) | 1.4 (1.4, 1.5) | 1.4 (1.4, 1.5) |
| Other | 4.6 (4.0, 5.3) | 2.2 (1.6, 2.7) | 1.8 (1.2, 2.4) | 1.5 (1.4, 1.7) | 1.5 (1.4, 1.7) | 1.5 (1.4, 1.7) |
| Unknown | 5.7 (4.3, 7.1) | 1.0 (-0.2, 2.3) | 0.6 (-0.7, 1.9) | 1.2 (0.9, 1.5) | 1.1 (0.8, 1.4) | 1.1 (0.8, 1.4) |
| All | 1.3 (1.3, 1.4) | 1.3 (1.3, 1.4) | 1.3 (1.3, 1.4) | 1.3 (1.3, 1.4) | 1.3 (1.3, 1.4) | 1.3 (1.3, 1.4) |
| **ASH medication, living arrangement** |  |  |  |  |  |  |
| Married | 4.0 (3.8, 4.1) | 4.5 (4.4, 4.6) | 4.8 (4.7, 5.0) | 5.5 (5.4, 5.6) | 5.5 (5.4, 5.6) | 5.5 (5.4, 5.6) |
| Cohabiting | 5.0 (4.8, 5.2) | 5.1 (4.9, 5.4) | 4.9 (4.7, 5.1) | 5.2 (5.0, 5.3) | 5.2 (5.0, 5.3) | 5.2 (5.0, 5.3) |
| Living alone | 8.7 (8.5, 9.0) | 8.2 (8.0, 8.5) | 7.8 (7.5, 8.0) | 5.8 (5.6, 5.9) | 5.8 (5.6, 5.9) | 5.8 (5.6, 5.9) |
| Other | 8.9 (8.2, 9.6) | 5.3 (4.7, 5.9) | 4.1 (3.5, 4.8) | 5.7 (5.3, 6.0) | 5.7 (5.4, 6.1) | 5.7 (5.4, 6.1) |
| Unknown | 8.9 (7.5, 10.3) | 2.1 (0.8, 3.5) | 0.7 (-0.6, 2.1) | 4.2 (3.5, 5.0) | 4.2 (3.4, 4.9) | 4.2 (3.4, 4.9) |
| All | 5.5 (5.4, 5.6) | 5.5 (5.4, 5.6) | 5.5 (5.4, 5.6) | 5.5 (5.4, 5.5) | 5.5 (5.4, 5.5) | 5.5 (5.4, 5.5) |

Notes: The probabilities were estimated by the margins command in Stata 14. 95% confidence intervals in brackets. ASH medication: anxiolytic/sedative/hypnotic medication. Anatomical Therapeutic Chemical (ATC) codes available in Table A. All analyses were controlled for 5-year age groups and year. Models were additionally controlled for educational attainment, economic activity, and number of children where mentioned.

**Results for logistic models**

**Figure A**―Odds ratios of psychotropic medication purchases for different living arrangements of men and women aged 25-39 years in 1995 followed up to 2007

|  |
| --- |
|  |
| \| **Logistic models Individual fixed effects models**  Model 1: Year and age  Model 2: Year, age, education, and economic activity  Model 3: Year, age, education, economic activity, and no. of children Model 3: Year and age \| **Individual fixed effects logistic models**  Model 4: Year and age  Model 5: Year, age, education, and economic activity  Model 6: Year, age, education, economic activity and no. of children \| \| --- \| --- \|   . |

Notes: The error bars represent 95% confidence intervals. All analyses were controlled for 5-year age groups and year. Models were additionally controlled for educational attainment, economic activity and number of children in the family, where mentioned. Full information on point estimates and 95% confidence intervals can be found in Table J.

**Figure B**―Odds ratios of psychotropic medication purchases for different living arrangements comparing parents with childless men and women aged 25-39 years in 1995 followed up to 2007

|  |
| --- |
|  |
| \| **Logistic models Individual fixed effects models**  Model 1: Year and age  Model 2: Year, age, education, and economic activity Model 3: Year and age \| **Individual fixed effects logistic models**  Model 3: Year and age  Model 4: Year, age, education, and economic activity \| \| --- \| --- \|   . |

Notes: The error bars represent 95% confidence intervals. All analyses were controlled for 5-year age groups and year. Models were additionally controlled for educational attainment, and economic activity, where mentioned. Men and women were defined as fathers and mothers respectively, when they had at least one child under the age of 18 years living in their family. Full information on point estimates and 95% confidence intervals can be found in Table K.

**Figure C**―Odds ratios of psychotropic medication purchases by subcategory for different living arrangements of men and women aged 25-39 years in 1995 followed up to 2007

|  |
| --- |
|  |
|  |
|  |
| \| **Logistic models Individual fixed effects models**  Model 1: Year and age  Model 2: Year, age, education, and economic activity  Model 3: Year, age, education, economic activity, and no. of children Model 3: Year and age \| **Individual fixed effects logistic models**  Model 4: Year and age  Model 5: Year, age, education, and economic activity  Model 6: Year, age, education, economic activity and no. of children \| \| --- \| --- \|   . |

Notes: The error bars represent 95% confidence intervals. All analyses were controlled for 5-year age groups and year. Models were additionally controlled for educational attainment, economic activity and number of children in the family, where mentioned. Full information on point estimates and 95% confidence intervals can be found in Table L.

**Table J**―Odds ratios of psychotropic medication purchases for different living arrangements of men and women aged 25-39 years in 1995, for 1995 to 2007

|  | **Logistic** | | | **Individual Fixed Effects Logistic** | | |
| --- | --- | --- | --- | --- | --- | --- |
|  | **Model 1** | **Model 2** | **Model 3** | **Model 4** | **Model 5** | **Model 6** |
| The models controlled for: | Year, age | Year, age, education, economic activity | Year, age, education, economic activity, no. of children | Year, age | Year, age, education, economic activity | Year, age, education, economic activity, no. of children |
| **Men, Sample size (No. of obs.)** | 63077 (800650) | 63077 (800650) | 63077 (800650) | 14360 (182685) | 14360 (182685) | 14360 (182685) |
| **Living arrangement** |  |  |  |  |  |  |
| Married | 0.83 (0.79, 0.88) | 0.94 (0.89, 1.00) | 1.04 (0.98, 1.11) | 1.20 (1.12, 1.27) | 1.20 (1.13, 1.28) | 1.22 (1.15, 1.30) |
| Cohabiting | Ref. | Ref. | Ref. | Ref. | Ref. | Ref. |
| Living alone | 2.41 (2.29, 2.55) | 1.86 (1.77, 1.97) | 1.64 (1.55, 1.74) | 1.26 (1.20, 1.34) | 1.25 (1.19, 1.33) | 1.21 (1.14, 1.29) |
| Other | 2.40 (2.24, 2.56) | 1.39 (1.30, 1.49) | 1.21 (1.13, 1.30) | 1.17 (1.09, 1.25) | 1.16 (1.08, 1.24) | 1.12 (1.04, 1.20) |
| Unknown | 3.21 (2.90, 3.55) | 1.10 (0.98, 1.23) | 0.96 (0.85, 1.08) | 0.89 (0.80, 0.99) | 0.87 (0.78, 0.96) | 0.84 (0.75, 0.93) |
| **Educational attainment** |  |  |  |  |  |  |
| Higher tertiary or more |  | Ref. | Ref. |  | Ref. | Ref. |
| Lower tertiary |  | 0.81 (0.74, 0.89) | 0.81 (0.74, 0.89) |  | 0.62 (0.49, 0.80) | 0.62 (0.49, 0.79) |
| Upper secondary |  | 0.81 (0.75, 0.88) | 0.81 (0.75, 0.88) |  | 0.75 (0.60, 0.93) | 0.74 (0.60, 0.93) |
| Compulsory |  | 0.90 (0.83, 0.98) | 0.90 (0.83, 0.98) |  | 0.96 (0.72, 1.29) | 0.96 (0.72, 1.29) |
| **Economic activity** |  |  |  |  |  |  |
| Employed |  | Ref. | Ref. |  | Ref. | Ref. |
| Unemployed |  | 2.43 (2.33, 2.54) | 2.41 (2.30, 2.52) |  | 1.12 (1.07, 1.18) | 1.12 (1.07, 1.18) |
| Students and pupils |  | 2.74 (2.54, 2.96) | 2.71 (2.51, 2.93) |  | 1.21 (1.11, 1.31) | 1.21 (1.11, 1.31) |
| Pensioners |  | 15.6 (14.57, 16.77) | 15.3 (14.28, 16.43) |  | 1.56 (1.44, 1.70) | 1.56 (1.43, 1.69) |
| Others |  | 2.49 (2.34, 2.65) | 2.45 (2.30, 2.61) |  | 1.28 (1.18, 1.38) | 1.27 (1.18, 1.37) |
| **No. of children < 18 years** |  |  |  |  |  |  |
| No children |  |  | Ref. |  |  | Ref. |
| 1 child |  |  | 1.25 (1.18, 1.32) |  |  | 1.10 (1.04, 1.17) |
| 2 children |  |  | 0.91 (0.86, 0.96) |  |  | 0.99 (0.94, 1.05) |
| 3 or more children |  |  | 0.88 (0.82, 0.95) |  |  | 1.06 (0.98, 1.15) |
| **Constant** | 0.319 (0.030, 0.034) | 0.028 (0.026, 0.313) | 0.026 (0.023, 0.029) | NA | NA | NA |
| **Women, Sample size (No. of obs.)** | 61101 (781368) | 61101 (781368) | 61101 (781368) | 19169 (246918) | 19169 (246918) | 19169 (246918) |
| **Living arrangement** |  |  |  |  |  |  |
| Married | 0.82 (0.78, 0.85) | 0.88 (0.84, 0.92) | 0.96 (0.92, 1.01) | 1.17 (1.11, 1.24) | 1.17 (1.11, 1.24) | 1.15 (1.09, 1.21) |
| Cohabiting | Ref. | Ref. | Ref. | Ref. | Ref. | Ref. |
| Living alone | 1.78 (1.70, 1.86) | 1.65 (1.58, 1.73) | 1.61 (1.54, 1.69) | 1.23 (1.17, 1.29) | 1.22 (1.17, 1.28) | 1.23 (1.17, 1.29) |
| Other | 2.13 (1.95, 2.32) | 1.14 (1.04, 1.26) | 1.01 (0.92, 1.11) | 1.18 (1.07, 1.29) | 1.17 (1.06, 1.28) | 1.18 (1.07, 1.29) |
| Unknown | 1.82 (1.55, 2.14) | 0.59 (0.50, 0.71) | 0.52 (0.44, 0.63) | 0.67 (0.57, 0.79) | 0.66 (0.56, 0.78) | 0.67 (0.57, 0.80) |
| **Educational attainment** |  |  |  |  |  |  |
| Higher tertiary or more |  | Ref. | Ref. |  | Ref. | Ref. |
| Lower tertiary |  | 0.92 (0.86, 0.98) | 0.92 (0.86, 0.98) |  | 1.04 (0.90, 1.21) | 1.05 (0.90, 1.22) |
| Upper secondary |  | 0.96 (0.90, 1.03) | 0.97 (0.91, 1.03) |  | 1.10 (0.94, 1.28) | 1.10 (0.94, 1.29) |
| Compulsory |  | 1.09 (1.02, 1.17) | 1.10 (1.02, 1.18) |  | 1.16 (0.95, 1.42) | 1.15 (0.94, 1.41) |
| **Economic activity** |  |  |  |  |  |  |
| Employed |  | Ref. | Ref. |  | Ref. | Ref. |
| Unemployed |  | 1.74 (1.68, 1.81) | 1.76 (1.69, 1.83) |  | 0.99 (0.94, 1.03) | 0.98 (0.94, 1.03) |
| Students and pupils |  | 1.62 (1.54, 1.71) | 1.64 (1.56, 1.74) |  | 1.02 (0.95, 1.08) | 1.01 (0.95, 1.08) |
| Pensioners |  | 13.89 (12.80, 15.08) | 13.23 (12.19, 14.36) |  | 1.82 (1.66, 1.99) | 1.82 (1.66, 2.00) |
| Other economic activity |  | 1.32 (1.26, 1.39) | 1.41 (1.35, 1.49) |  | 1.00 (0.95, 1.06) | 1.00 (0.94, 1.05) |
| **No. of children < 18 years** |  |  |  |  |  |  |
| No children |  |  | 1.16 (1.11, 1.21) |  |  | 0.98 (0.93, 1.02) |
| 1 child |  |  | Ref. |  |  | Ref. |
| 2 children |  |  | 0.86 (0.83, 0.90) |  |  | 1.11 (1.06, 1.16) |
| 3 or more children |  |  | 0.77 (0.73, 0.81) |  |  | 1.18 (1.11, 1.26) |
| **Constant** | 0.047 (0.044, 0.050) | 0.040 (0.037, 0.043) | 0.038 (0.034, 0.041) | NA | NA | NA |

Notes*:* Ref.: reference category. 95% confidence intervals in brackets. All analyses were controlled for 5-year age groups and year. Models were additionally controlled for educational attainment, economic activity, and number of children in the family where mentioned. The ‘other economic activity’ included the categories other, unknown, conscripts, and conscientious objectors.

**Table K**―Odds ratios of psychotropic medication purchases for different living arrangements comparing parents with childless men and women aged 25-39 years in 1995, for 1995 to 2007

|  | **Logistic** | | **Individual Fixed Effects Logistic** | |
| --- | --- | --- | --- | --- |
|  | **Model 1** | **Model 2** | **Model 3** | **Model 4** |
| The model controlled for: | Year, age | Year, age, education, economic activity | Year, age | Year, age, education, economic activity |
| **Men** |  |  |  |  |
| **Childless men** |  |  |  |  |
| Sample size (No. of obs.) | 46350 (381956) | 46350 (381956) | 8533 (89319) | 8533 (89319) |
| **Living arrangement** |  |  |  |  |
| Married | 0.98 (0.90, 1.07) | 1.10 (1.01, 1.20) | 1.17 (1.05, 1.30) | 1.17 (1.05, 1.31) |
| Cohabiting | Ref. | Ref. | Ref. | Ref. |
| Living alone | 2.11 (1.98, 2.26) | 1.70 (1.59, 1.82) | 1.24 (1.15, 1.33) | 1.23 (1.14, 1.33) |
| Other | 2.01 (1.87, 2.17) | 1.22 (1.13, 1.32) | 1.15 (1.05, 1.25) | 1.14 (1.05, 1.25) |
| Unknown | 0.98 (0.90, 1.07) | 1.10 (1.01, 1.20) | 1.17 (1.05, 1.30) | 1.17 (1.05, 1.31) |
| **Fathers** |  |  |  |  |
| Sample size (No. of obs.) | 44492 (418694) | 44492 (418694) | 6904 (70801) | 6904 (70801) |
| **Living arrangement** |  |  |  |  |
| Married | 0.90 (0.84, 0.97) | 0.96 (0.90, 1.04) | 1.33 (1.18, 1.50) | 1.32 (1.17, 1.49) |
| Cohabiting | Ref. | Ref. | Ref. | Ref. |
| Living alone | 1.50 (1.31, 1.71) | 1.35 (1.18, 1.54) | 1.04 (0.88, 1.23) | 1.02 (0.86, 1.21) |
| Other | 2.57 (1.79, 3.69) | 1.78 (1.22, 2.59) | 1.63 (0.79, 3.39) | 1.68 (0.80, 3.50) |
| Unknown | NA | NA | NA | NA |
| **Comparing parenthood status by living arrangement – p value** |  |  |  |  |
| **Living arrangement** |  |  |  |  |
| Married | 0.122 | **0.023** | 0.112 | 0.147 |
| Cohabiting |  |  |  |  |
| Living alone | **<0.001** | **0.002** | **0.063** | **0.043** |
| Other | 0.194 | **0.055** | 0.349 | 0.309 |
| Unknown | NA | NA | NA | NA |
| **Women** |  |  |  |  |
| **Childless women** |  |  |  |  |
| Sample size (No. of obs.) | 35534 (255638) | 35534 (255638) | 7432 (73205) | 7432 (73205) |
| **Living arrangement** |  |  |  |  |
| Married | 0.89 (0.83, 0.96) | 0.96 (0.89, 1.03) | 1.10 (1.00, 1.22) | 1.10 (1.00, 1.22) |
| Cohabiting | Ref. | Ref. | Ref. | Ref. |
| Living alone | 1.68 (1.58, 1.79) | 1.56 (1.46, 1.67) | 1.24 (1.15, 1.33) | 1.23 (1.15, 1.33) |
| Other | 1.72 (1.57, 1.89) | 0.94 (0.85, 1.04) | 1.19 (1.07, 1.32) | 1.17 (1.05, 1.31) |
| Unknown | 1.45 (1.23, 1.71) | 0.48 (0.40, 0.57) | 0.64 (0.53, 0.78) | 0.62 (0.51, 0.75) |
| **Mothers** |  |  |  |  |
| Sample size (No. of obs.) | 49431 (525730) | 49431 (525730) | 12956 (146763) | 12956 (146763) |
| **Living arrangement** |  |  |  |  |
| Married | 0.91 (0.86, 0.96) | 0.94 (0.89, 1.00) | 1.24 (1.15, 1.33) | 1.23 (1.14, 1.33) |
| Cohabiting | Ref. | Ref. | Ref. | Ref. |
| Living alone | 1.76 (1.66, 1.87) | 1.68 (1.58, 1.78) | 1.22 (1.14, 1.31) | 1.22 (1.14, 1.31) |
| Other | 2.33 (1.15, 4.74) | 1.29 (0.65, 2.55) | 1.56 (0.44, 5.49) | 1.46 (0.42, 5.12) |
| Unknown | NA | NA | NA | NA |
| **Comparing parenthood status by living arrangement – p value** |  |  |  |  |
| **Living arrangement** |  |  |  |  |
| Married | 0.711 | 0.721 | 0.084 | 0.089 |
| Cohabiting |  |  |  |  |
| Living alone | 0.314 | 0.106 | 0.821 | 0.834 |
| Other | 0.407 | 0.369 | 0.675 | 0.734 |
| Unknown | NA | NA | NA | NA |

Notes: Ref.: reference category. 95% confidence intervals in brackets. All analyses were controlled for 5-year age groups and year. Models were additionally controlled for educational attainment and economic activity where mentioned. Men and women were defined as fathers and mothers respectively, when they had at least one child under the age of 18 years living in their family. NA: Not available, as for individuals in the unknown category, information on children was not available.

**Table L**―Odds ratios of psychotropic medication purchases by subcategory for different living arrangements of men and women aged 25-39 years in 1995, for 1995 to 2007

|  | **Logistic** | | | **Individual Fixed Effects Logistic** | | |
| --- | --- | --- | --- | --- | --- | --- |
|  | **Model 1** | **Model 2** | **Model 3** | **Model 4** | **Model 5** | **Model 6** |
| The models controlled for: | Year, age | Year, age, education, economic activity | Year, age, education, economic activity, no. of children | Year, age | Year, age, education, economic activity | Year, age, education, economic activity, no. of children |
| **Men** |  |  |  |  |  |  |
| **All psychotropic medication, living arrangement** | |  |  |  |  |  |
| Sample size (No. of obs.) | 63077 (800650) | 63077 (800650) | 63077 (800650) | 14360 (182685) | 14360 (182685) | 14360 (182685) |
| Married | 0.83 (0.79, 0.88) | 0.94 (0.89, 1.00) | 1.04 (0.98, 1.11) | 1.20 (1.12, 1.27) | 1.20 (1.13, 1.28) | 1.22 (1.15, 1.30) |
| Cohabiting | Ref. | Ref. | Ref. | Ref. | Ref. | Ref. |
| Living alone | 2.41 (2.29, 2.55) | 1.86 (1.77, 1.97) | 1.64 (1.55, 1.74) | 1.26 (1.20, 1.34) | 1.25 (1.19, 1.33) | 1.21 (1.14, 1.29) |
| Other | 2.40 (2.24, 2.56) | 1.39 (1.30, 1.49) | 1.21 (1.13, 1.30) | 1.17 (1.09, 1.25) | 1.16 (1.08, 1.24) | 1.12 (1.04, 1.20) |
| Unknown | 3.21 (2.90, 3.55) | 1.10 (0.98, 1.23) | 0.96 (0.85, 1.08) | 0.89 (0.80, 0.99) | 0.87 (0.78, 0.96) | 0.84 (0.75, 0.93) |
| **Antidepressants, living arrangement** |  |  |  |  |  |  |
| Sample size (No. of obs.) | 63077 (800650) | 63077 (800650) | 63077 (800650) | 10416 (132347) | 10416 (132347) | 10416 (132347) |
| Married | 0.90 (0.84, 0.96) | 0.99 (0.93, 1.06) | 1.05 (0.98, 1.13) | 1.31 (1.22, 1.41) | 1.31 (1.22, 1.42) | 1.27 (1.18, 1.37) |
| Cohabiting | Ref. | Ref. | Ref. | Ref. | Ref. | Ref. |
| Living alone | 2.23 (2.09, 2.38) | 1.77 (1.66, 1.88) | 1.63 (1.52, 1.74) | 1.19 (1.11, 1.27) | 1.18 (1.11, 1.26) | 1.22 (1.14, 1.31) |
| Other | 1.79 (1.65, 1.94) | 1.15 (1.06, 1.25) | 1.05 (0.96, 1.15) | 1.12 (1.03, 1.22) | 1.11 (1.02, 1.20) | 1.15 (1.05, 1.25) |
| Unknown | 2.13 (1.88, 2.41) | 0.93 (0.81, 1.06) | 0.85 (0.74, 0.97) | 1.00 (0.88, 1.13) | 0.98 (0.86, 1.11) | 1.01 (0.89, 1.15) |
| **Antipsychotics, living arrangement** |  |  |  |  |  |  |
| Sample size (No. of obs.) | 63077 (800650) | 63077 (800650) | 63077 (800650) | 3130 (38743) | 3130 (38743) | 3130 (38743) |
| Married | 0.72 (0.63, 0.83) | 0.93 (0.81, 1.07) | 1.15 (1.00, 1.34) | 1.09 (0.93, 1.28) | 1.08 (0.92, 1.27) | 1.09 (0.92, 1.29) |
| Cohabiting | Ref. | Ref. | Ref. | Ref. | Ref. | Ref. |
| Living alone | 5.24 (4.66, 5.90) | 3.09 (2.73, 3.48) | 2.41 (2.13, 2.74) | 1.40 (1.23, 1.60) | 1.35 (1.19, 1.54) | 1.29 (1.13, 1.49) |
| Other | 7.31 (6.44, 8.31) | 2.78 (2.43, 3.18) | 2.14 (1.86, 2.46) | 1.28 (1.10, 1.48) | 1.23 (1.06, 1.43) | 1.18 (1.01, 1.38) |
| Unknown | 11.37 (9.61, 13.45) | 2.17 (1.81, 2.61) | 1.67 (1.38, 2.02) | 0.99 (0.82, 1.20) | 0.90 (0.74, 1.09) | 0.86 (0.71, 1.05) |
| **Antimanic agents, living arrangement** |  |  |  |  |  |  |
| Sample size (No. of obs.) | 63077 (800650) | 63077 (800650) | 63077 (800650) | 1995 (24864) | 1995 (24864) | 1995 (24864) |
| Married | 0.74 (0.63, 0.88) | 0.88 (0.74, 1.05) | 1.05 (0.87, 1.25) | 0.90 (0.72, 1.11) | 0.90 (0.73, 1.12) | 1.01 (0.81, 1.27) |
| Cohabiting | Ref. | Ref. | Ref. | Ref. | Ref. | Ref. |
| Living alone | 2.66 (2.28, 3.10) | 1.63 (1.39, 1.91) | 1.34 (1.13, 1.58) | 1.16 (0.97, 1.38) | 1.12 (0.94, 1.34) | 0.99 (0.82, 1.20) |
| Other | 3.88 (3.27, 4.60) | 1.69 (1.42, 2.02) | 1.37 (1.14, 1.64) | 1.00 (0.82, 1.22) | 0.96 (0.79, 1.17) | 0.85 (0.69, 1.05) |
| Unknown | 5.17 (4.15, 6.44) | 1.28 (1.02, 1.61) | 1.04 (0.82, 1.32) | 0.88 (0.69, 1.13) | 0.83 (0.65, 1.06) | 0.73 (0.56, 0.94) |
| **ASH medication, living arrangement** |  |  |  |  |  |  |
| Sample size (No. of obs.) | 63077 (800650) | 63077 (800650) | 63077 (800650) | 10207 (129164) | 10207 (129164) | 10207 (129164) |
| Married | 0.81 (0.75, 0.86) | 0.93 (0.87, 0.99) | 1.05 (0.98, 1.13) | 1.17 (1.08, 1.26) | 1.17 (1.08, 1.26) | 1.22 (1.12, 1.31) |
| Cohabiting | Ref. | Ref. | Ref. | Ref. | Ref. | Ref. |
| Living alone | 2.37 (2.22, 2.53) | 1.82 (1.71, 1.94) | 1.57 (1.46, 1.68) | 1.31 (1.23, 1.40) | 1.30 (1.22, 1.39) | 1.23 (1.15, 1.32) |
| Other | 1.99 (1.84, 2.15) | 1.19 (1.10, 1.29) | 1.01 (0.93, 1.10) | 1.20 (1.11, 1.31) | 1.20 (1.10, 1.30) | 1.12 (1.03, 1.22) |
| Unknown | 3.24 (2.90, 3.63) | 1.25 (1.10, 1.41) | 1.06 (0.94, 1.20) | 1.08 (0.97, 1.22) | 1.07 (0.95, 1.20) | 1.00 (0.89, 1.13) |
| **Women** |  |  |  |  |  |  |
| **All psychotropic medication, living arrangement** | |  |  |  |  |  |
| Sample size (No. of obs.) | 61101 (781368) | 61101 (781368) | 61101 (781368) | 19169 (246918) | 19169 (246918) | 19169 (246918) |
| Married | 0.82 (0.78, 0.85) | 0.88 (0.84, 0.92) | 0.96 (0.92, 1.01) | 1.17 (1.11, 1.24) | 1.17 (1.11, 1.24) | 1.15 (1.09, 1.21) |
| Cohabiting | Ref. | Ref. | Ref. | Ref. | Ref. | Ref. |
| Living alone | 1.78 (1.70, 1.86) | 1.65 (1.58, 1.73) | 1.61 (1.54, 1.69) | 1.23 (1.17, 1.29) | 1.22 (1.17, 1.28) | 1.23 (1.17, 1.29) |
| Other | 2.13 (1.95, 2.32) | 1.14 (1.04, 1.26) | 1.01 (0.92, 1.11) | 1.18 (1.07, 1.29) | 1.17 (1.06, 1.28) | 1.18 (1.07, 1.29) |
| Unknown | 1.82 (1.55, 2.14) | 0.59 (0.50, 0.71) | 0.52 (0.44, 0.63) | 0.67 (0.57, 0.79) | 0.66 (0.56, 0.78) | 0.67 (0.57, 0.80) |
| **Antidepressants, living arrangement** |  |  |  |  |  |  |
| Sample size (No. of obs.) | 61101 (781368) | 61101 (781368) | 61101 (781368) | 14556 (187524) | 14556 (187524) | 14556 (187524) |
| Married | 0.85 (0.81, 0.90) | 0.90 (0.86, 0.95) | 0.95 (0.90, 1.01) | 1.24 (1.16, 1.32) | 1.24 (1.16, 1.31) | 1.18 (1.10, 1.25) |
| Cohabiting | Ref. | Ref. | Ref. | Ref. | Ref. | Ref. |
| Living alone | 1.73 (1.65, 1.83) | 1.61 (1.53, 1.70) | 1.59 (1.51, 1.68) | 1.17 (1.11, 1.24) | 1.18 (1.11, 1.24) | 1.18 (1.12, 1.25) |
| Other | 1.39 (1.26, 1.54) | 0.85 (0.76, 0.95) | 0.80 (0.72, 0.90) | 1.16 (1.04, 1.29) | 1.16 (1.04, 1.29) | 1.20 (1.08, 1.34) |
| Unknown | 1.22 (1.01, 1.47) | 0.55 (0.46, 0.67) | 0.52 (0.43, 0.63) | 0.85 (0.71, 1.03) | 0.84 (0.70, 1.01) | 0.90 (0.75, 1.09) |
| **Antipsychotics, living arrangement** |  |  |  |  |  |  |
| Sample size (No. of obs.) | 61101 (781368) | 61101 (781368) | 61101 (781368) | 2819 (35837) | 2819 (35837) | 2819 (35837) |
| Married | 0.63 (0.55, 0.71) | 0.78 (0.68, 0.88) | 1.02 (0.89, 1.16) | 1.00 (0.86, 1.17) | 1.01 (0.87, 1.18) | 1.05 (0.90, 1.22) |
| Cohabiting | Ref. | Ref. | Ref. | Ref. | Ref. | Ref. |
| Living alone | 2.55 (2.28, 2.86) | 2.07 (1.84, 2.32) | 1.94 (1.72, 2.18) | 1.29 (1.13, 1.46) | 1.28 (1.13, 1.46) | 1.28 (1.13, 1.46) |
| Other | 6.39 (5.47, 7.46) | 1.82 (1.52, 2.18) | 1.36 (1.14, 1.63) | 1.17 (0.96, 1.43) | 1.16 (0.95, 1.41) | 1.13 (0.93, 1.38) |
| Unknown | 6.41 (5.09, 8.07) | 1.01 (0.78, 1.30) | 0.75 (0.59, 0.97) | 0.68 (0.52, 0.90) | 0.63 (0.47, 0.83) | 0.61 (0.46, 0.81) |
| **Antimanic agents, living arrangement** |  |  |  |  |  |  |
| Sample size (No. of obs.) | 61101 (781368) | 61101 (781368) | 61101 (781368) | 1934 (24753) | 1934 (24753) | 1934 (24753) |
| Married | 0.72 (0.62, 0.84) | 0.86 (0.74, 1.00) | 1.01 (0.86, 1.18) | 1.17 (0.96, 1.43) | 1.18 (0.96, 1.44) | 1.15 (0.94, 1.40) |
| Cohabiting | Ref. | Ref. | Ref. | Ref. | Ref. | Ref. |
| Living alone | 1.92 (1.66, 2.21) | 1.50 (1.30, 1.74) | 1.44 (1.25, 1.66) | 1.43 (1.22, 1.68) | 1.40 (1.19, 1.65) | 1.41 (1.20, 1.66) |
| Other | 4.79 (3.94, 5.82) | 1.57 (1.28, 1.92) | 1.32 (1.08, 1.62) | 1.57 (1.24, 2.00) | 1.55 (1.22, 1.97) | 1.58 (1.24, 2.01) |
| Unknown | 5.46 (4.11, 7.24) | 1.15 (0.86, 1.54) | 0.97 (0.72, 1.30) | 1.12 (0.81, 1.56) | 1.08 (0.78, 1.51) | 1.10 (0.79, 1.54) |
| **ASH medication, living arrangement** |  |  |  |  |  |  |
| Sample size (No. of obs.) | 61101 (781368) | 61101 (781368) | 61101 (781368) | 12893 (165683) | 12893 (165683) | 12893 (165683) |
| Married | 0.79 (0.75, 0.84) | 0.86 (0.81, 0.91) | 0.97 (0.92, 1.03) | 1.12 (1.05, 1.20) | 1.12 (1.05, 1.20) | 1.14 (1.06, 1.22) |
| Cohabiting | Ref. | Ref. | Ref. | Ref. | Ref. | Ref. |
| Living alone | 1.83 (1.73, 1.94) | 1.67 (1.58, 1.77) | 1.62 (1.53, 1.72) | 1.16 (1.10, 1.23) | 1.16 (1.09, 1.23) | 1.16 (1.09, 1.23) |
| Other | 1.97 (1.78, 2.19) | 1.11 (1.00, 1.24) | 0.96 (0.86, 1.07) | 1.18 (1.06, 1.31) | 1.18 (1.06, 1.31) | 1.16 (1.04, 1.29) |
| Unknown | 1.92 (1.59, 2.31) | 0.74 (0.61, 0.90) | 0.64 (0.52, 0.77) | 0.84 (0.70, 1.01) | 0.84 (0.70, 1.00) | 0.81 (0.68, 0.97) |

Notes: Ref.: reference category. 95% confidence intervals in brackets. ASH medication: anxiolytic/sedative/hypnotic medication. Anatomical Therapeutic Chemical (ATC) codes available in Table A. All analyses were controlled for 5-year age groups and year. Models were additionally controlled for educational attainment, economic activity, and number of children where mentioned.
